# Hydrocarbon Desaturation in Cyanobacterial Thylakoid Membranes Is Linked With Acclimation to Suboptimal Growth Temperatures

**DOI:** 10.3389/fmicb.2021.781864

**Published:** 2021-11-26

**Authors:** Eerika Vuorio, Kati Thiel, Duncan Fitzpatrick, Tuomas Huokko, Jari Kämäräinen, Hariharan Dandapani, Eva-Mari Aro, Pauli Kallio

**Affiliations:** Molecular Plant Biology, Department of Life Technologies, University of Turku, Turku, Finland

**Keywords:** cyanobacteria, *Synechocystis* sp. PCC 6803, hydrocarbon saturation, alkane, alkene, membrane fluidity, aldehyde deformylating oxygenase, medium chain-length aliphatic

## Abstract

The ability to produce medium chain length aliphatic hydrocarbons is strictly conserved in all photosynthetic cyanobacteria, but the molecular function and biological significance of these compounds still remain poorly understood. This study gives a detailed view to the changes in intracellular hydrocarbon chain saturation in response to different growth temperatures and osmotic stress, and the associated physiological effects in the model cyanobacterium *Synechocystis* sp. PCC 6803. We show that the ratio between the representative hydrocarbons, saturated heptadecane and desaturated heptadecene, is reduced upon transition from 38°C toward 15°C, while the total content is not much altered. In parallel, it appears that in the hydrocarbon-deficient ∆*ado* (aldehyde deformylating oxygenase) mutant, phenotypic and metabolic changes become more evident under suboptimal temperatures. These include hindered growth, accumulation of polyhydroxybutyrate, altered pigment profile, restricted phycobilisome movement, and ultimately reduced CO_2_ uptake and oxygen evolution in the ∆*ado* strain as compared to *Synechocystis* wild type. The hydrocarbons are present in relatively low amounts and expected to interact with other nonpolar cellular components, including the hydrophobic part of the membrane lipids. We hypothesize that the function of the aliphatic chains is specifically associated with local fluidity effects of the thylakoid membrane, which may be required for the optimal movement of the integral components of the photosynthetic machinery. The findings support earlier studies and expand our understanding of the biological role of aliphatic hydrocarbons in acclimation to low temperature in cyanobacteria and link the proposed role in the thylakoid membrane to changes in photosynthetic performance, central carbon metabolism, and cell growth, which need to be effectively fine-tuned under alternating conditions in nature.

## Introduction

Unlike prokaryotes in general, all known photosynthetic cyanobacteria harbor the capacity to produce aliphatic hydrocarbons in their native metabolism (Coates et al., 2014; Lea-Smith et al., 2015). The products are hydrophobic and biochemically inert linear or methylated branched alkanes and alkenes, with an average carbon chain length between C15 and C19 (Coates et al., 2014). In general, apart from serving as an external source of carbon and energy for certain hydrocarbon-utilizing microbes (Xu et al., 2018), these compounds do not have any common established role in cell metabolism, and they are not typical constituents of biological membranes in nature. Some higher organisms produce aliphatic hydrocarbons as protective structural components, and they are found for example in plant leaves (Eglinton et al., 1962) and in feathers of certain birds (Jacob, 1978), while certain insects utilize hydrocarbons also for chemical signaling (Blomquist and Ginzel, 2021). Although the presence of hydrocarbons could affect cellular properties, such as resistance to desiccation or buoyancy in aquatic environments (Coates et al., 2014; Valentine and Reddy, 2015), their fundamental biological purpose and the underlying functional mechanisms in cyanobacteria still remain elusive. Based on the mere physicochemical properties, the mid-chain length hydrocarbons are bound to interact with other nonpolar components of the cell, with a potential consequent impact on the molecular organization and function of lipid bilayers. In support of this, hydrocarbons have been found in the cyanobacterial lipid fractions and specifically as part of the thylakoid membranes (Lea-Smith et al., 2016), which harbor the photosynthetic apparatus and associated protein complexes. In this context, hydrocarbons have been reported to serve as structural components that stack together and enhance the thylakoid membrane curvature (Lea-Smith et al., 2016), which again may have an influence on the topology, movement, and dynamic interplay of the embedded integral proteins. Intriguingly, despite the explicit conservation throughout evolution, hydrocarbons have not been found to be essential for cyanobacteria under any tested laboratory conditions, and the biosynthetic pathways can be disrupted without compromising the viability of the cells: Inactivation of hydrocarbon biosynthesis has been reported to result in reduced growth (Berla et al., 2015; Lea-Smith et al., 2016) and defected cell division under photoautotrophic conditions (Lea-Smith et al., 2016) in the cyanobacterial model strains *Synechocystis* sp. PCC 6803 (*Synechocystis* from here on) and *Synechococcus* sp. PCC 7002 (*Synechococcus* 7002 from here on). As expected based on the thylakoid association, this has been accompanied by changes in the photosynthetic functions, although the direct and indirect relationships are not always clear. The observed effects include enhanced cyclic electron flow as recorded under reduced temperatures, possibly in response changes in the intracellular ATP:NADPH ratio (Berla et al., 2015), and alterations in the photosystem (PS) II/I ratio, phycobilisome coupling, and oxygen uptake, without any significant changes on the net O_2_ evolution (Lea-Smith et al., 2016).

**Figure 1:**
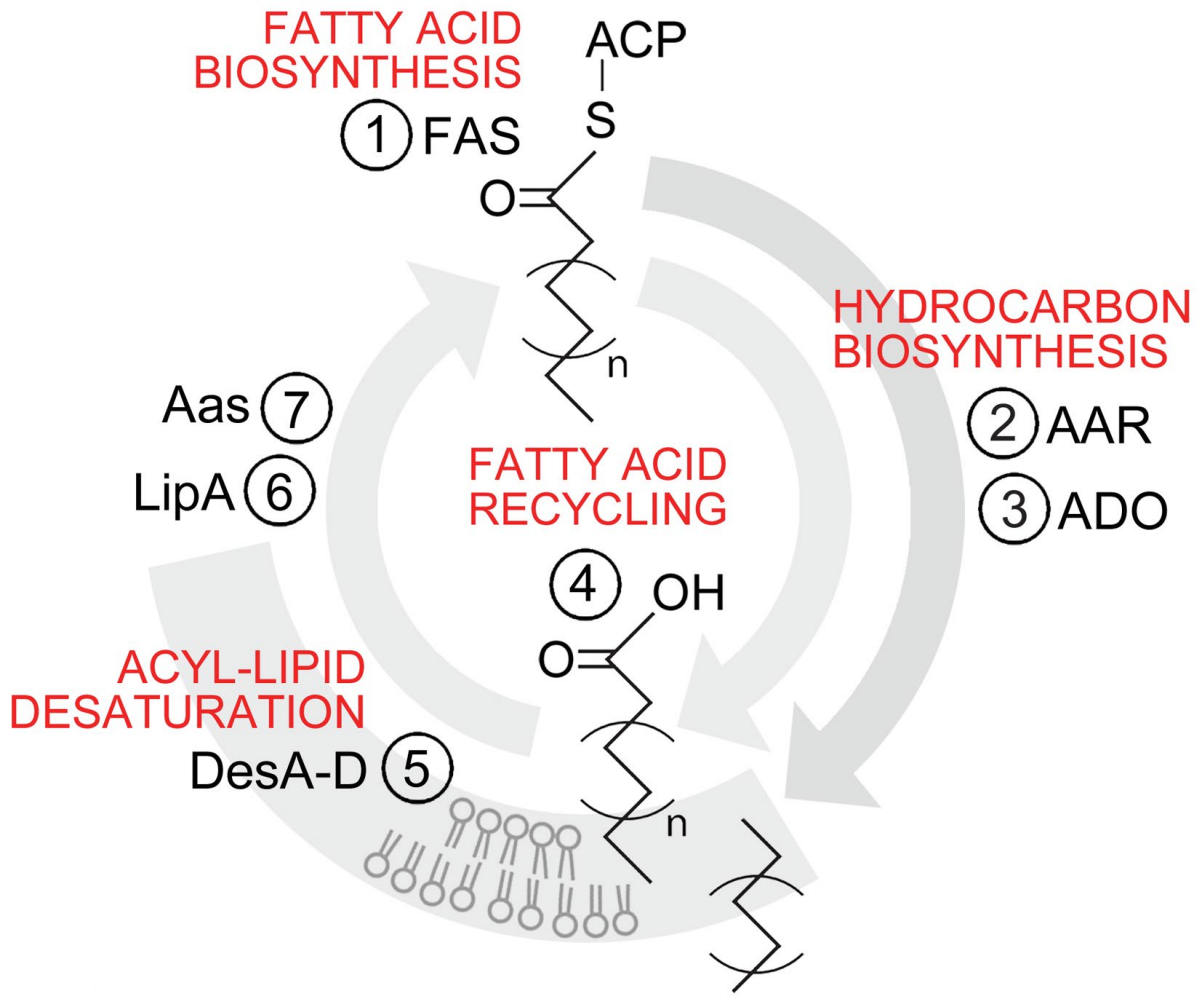
Biosynthesis of saturated and unsaturated aliphatic hydrocarbons *via* the AAR/ADO pathway in *Synechocystis* sp. PCC 6803. The (1) fatty acid synthase (FAS) fatty acyl – acyl carrier protein (ACP) serves as the precursor for hydrocarbon biosynthesis. In this process, the fatty acids are first released from ACP and reduced into aldehydes by the (2) fatty acyl-ACP reductase (AAR), followed by the conversion into corresponding C_n−1_ alkanes or alkenes by the (3) aldehyde deformylating oxygenase (ADO). The product specificity is determined by the length and saturation level of the initial acyl chain, and the hydrocarbons that derive from *de novo* fatty acid biosynthesis are exclusively linear saturated alkanes. When (4) fatty acids are used as structural components for phospholipid bilayer biosynthesis, the resulting glycerol-linked fatty acyl moieties in the membranes can be unsaturated by (5) the desaturases DesA-D. These products can be released from the membranes by the (6) lipolytic enzyme (LipA), and re-attached to ACP by (7) acyl-ACP synthetase (Aas), which may again serve as precursors for AAR/ADO to produce alkenes. The “n” represents the number of repeating two-carbon units in the carbon chain, and for heptadecane and heptadecene, “*n*=7.”

Since their discovery, the cyanobacterial hydrocarbon biosynthetic pathways have been extensively studied from the perspective of biotechnological applications. The motivation has been to establish production systems for renewable aliphatic hydrocarbons, as replacements for corresponding petroleum-derived chemicals, including biofuels, solvents, and precursors for plastic industry. Despite the relatively high estimated global production levels in nature (Lea-Smith et al., 2015), the hydrocarbon levels in cyanobacterial cells are typically maintained low (~0.1% of dry biomass; Tan et al., 2011; Coates et al., 2014), and efforts to engineer more efficient over-production strains have proven challenging (Kaczmarzyk and Fulda, 2010; Hu et al., 2013; Wang et al., 2013). This may, in part, reflect the unoptimal kinetic properties of the associated enzymes (Andre et al., 2013; Khara et al., 2013; Kallio et al., 2014; Bao et al., 2016), but also the complex native regulatory systems (Klähn et al., 2014) and the corresponding upstream mechanisms that control the intracellular hydrocarbon content, which have not been elucidated. There are two alternative, mutually exclusive hydrocarbon biosynthetic pathways found in all sequenced cyanobacterial species, the acyl-ACP reductase/aldehyde deformylating oxygenase (AAR/ADO) pathway (Schirmer et al., 2010; Coates et al., 2014; Lea-Smith et al., 2015) and the olefin synthase (Ols) pathway (Mendez-Perez et al., 2011; Coates et al., 2014; Lea-Smith et al., 2015). Most cyanobacteria, including *Synechocystis*, harbor the AAR/ADO pathway (Coates et al., 2014; Figure 1), which is the focus of this work. In this pathway, the enzyme fatty acyl-ACP reductase (AAR encoded by *sll0209*; *aar*) first releases a fatty acyl intermediate from the acyl carrier protein (ACP), followed by reduction into the corresponding fatty aldehyde. In the closely coupled subsequent step (Chang et al., 2020), the enzyme aldehyde deformylating oxygenase (ADO encoded by *sll0208*; *ado*) catalyzes the oxygen-dependent conversion (Li et al., 2012) of the aldehyde into the final linear C_n−1_ hydrocarbon product, simultaneously releasing formate as a side product (Warui et al., 2011). The hydrocarbons synthesized *de novo via* the endogenous fatty acid biosynthesis machinery are exclusively saturated alkanes, heptadecane being the predominant observed product (Coates et al., 2014). *Synechocystis* is also able to produce unsaturated hydrocarbon chains through the action of different desaturase enzymes. All four desaturases in *Synechocystis*, DesA-D, are acyl-lipid type desaturases which can generate double bonds exclusively on fatty acyl moieties linked with the glycerol backbone of membrane lipids (Murata and Wada, 1995). Subsequently, the acyl chains can be released by the action of the lipolytic enzyme (LipA encoded by *sll1969*) and re-attached to the ACP by acyl-ACP synthetase (Aas encoded by *slr1609*), which may again serve as a substrate for the AAR/ADO. Therefore, all the alkenes produced by *Synechocystis* derive from the membranes, heptadecene being the primary reported product. Although the different catalytic enzyme functions have been elucidated, the regulation of the alternative pathways and thus the production of different hydrocarbons in response to environmental changes is currently not understood.

The initiative to this study was to expand our knowledge on the conditional regulation of cyanobacterial hydrocarbon biosynthesis, which could also shed light on the biological role, and associated selective advantage underlying the evolutionary conservation of this trait. To this end, we first evaluated the effect of temperature and osmotic pressure on hydrocarbon production profiles in *Synechocystis*, to pinpoint conditions which specifically trigger qualitative and quantitative changes in the hydrocarbon composition. Next, the hydrocarbon-deficient ∆*ado* mutant was compared to *Synechocystis* wild-type (WT) to see how the hydrocarbon production patterns align with the expression and localization of the key enzyme ADO and to evaluate whether the regulation takes place mainly at the precursor level or at the level of AAR/ADO expression. This also allowed us to further study the connection between hydrocarbon accumulation and phenotypic cellular properties, including growth, gas fluxes, photosynthetic fluorescent parameters, and storage compound accumulation.

## Materials and Methods

### General Molecular Biology Methods

Standard molecular biology procedures and commercial kits (Qiagen) were used for DNA plasmid isolation and manipulation. Enzymes were purchased from New England BioLabs or from Thermo Fischer Scientific. Oligonucleotides were ordered from Eurofins MWG Operon. The chemicals used in the study were purchased from Sigma-Aldrich, if not mentioned otherwise.

### Organisms and Standard Growth Conditions

*Synechocystis* (Kaplan) was grown in BG-11 medium (Rippka et al., 1979) buffered with HEPES-NaOH to pH 7.5. BG-11 plates containing 1.5% agar were maintained under 1% CO_2_ in a Sanyo growth chambers, and liquid pre-cultures were cultivated with atmospheric carbon dioxide and 120rpm rotation in Algaetron growth chambers (Photon Systems Instruments) at 30°C. Main cultures were grown in MC1000 photobioreactor (Photon Systems Instruments) equipped with a cooling system (Hailea) under atmospheric CO_2_ at 15, 22, 30, and 38°C for 4–12days with or without supplemented 0.5M sorbitol. The lights were always calibrated to 50μmolm^−2^ s^−1^ with Li-COR (Li-250A Light Meter, Biosciences). *Escherichia coli* strain DH5α, used as the host for the plasmid construction, was grown in Luria-Bertani (LB) medium at 37°C on a shaker at 200rpm or on the solid agar plates supplemented with 50μg/ml kanamycin antibiotic.

### Construction of *Synechocystis* Deletion Mutant for ADO

A DNA plasmid construct was designed for the disruption of the gene *sll0208* coding for ADO in *Synechocystis* by integration of a kanamycin resistance cassette (Km^R^) in the middle of the gene by a homologous recombination. The DNA fragment was PCR-amplified using *Synechocystis* genomic DNA as template and Fwd_*sll0208*-*Sac*I and Rev_ *sll0208*-*BamH*I (Table S1) as the cloning primers. The resulting DNA was subcloned into the *Sac*I-*BamH*I -site of pUC19 (New England BioLabs) and disrupted by inserting a kanamycin (Km) resistance cassette (Km^R^; encoding aminoglycoside 3′-phosphotransferase in *Corynebacterium diphtheria*). The Km^R^ was amplified from pCOLA-Duet-1 (Novagen) using primers Km^R^-*Nco*I-for and Km^R^-*Nco*I-rev (Table S1). The generated pUC19-*sll0208*::Km^R^ was amplified in *E. coli* and used for natural transformation (Eaton-Rye, 2011) WT *Synechocystis* to obtain the strain Δ*ado*. To confirm segregation, positive colonies were re-streaked several times on BG-11 plates supplemented with increasing amount of kanamycin and analyzed by colony PCR.

### Preparation of Samples for Small-Scale Hydrocarbon Detection From Whole Cells

**Figure 2:**
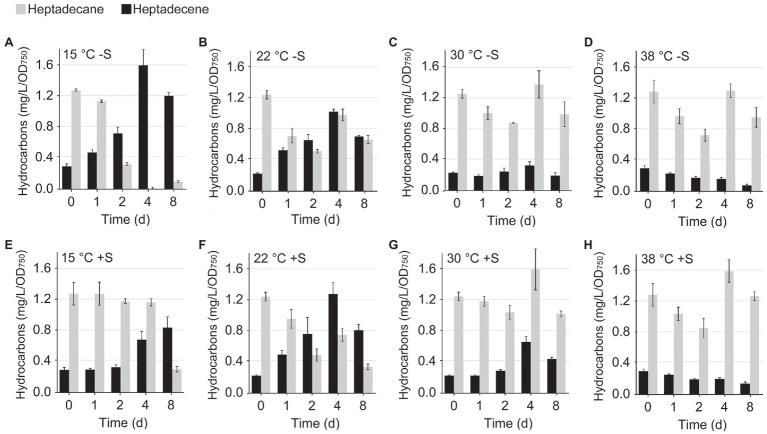
Accumulation of intracellular heptadecene (black bars) and heptadecane (grey bars) in *Synechocystis* sp. PCC 6803 cells cultivated under different temperatures in standard BG-11 (-S) and in the presence of supplemented 0.5M sorbitol (+S). The production was monitored over eight consecutive days under **(A)** 15°C -S, **(B)** 22°C -S, **(C)** 30°C -S, **(D)** 38°C -S, **(E)** 15°C+S, **(F)** 22°C+S **(G)** 30°C+S, and **(H)** 38°C+S. The averages and standard deviations were calculated based on three parallel independent cultures. See Supplementary Figure S5 for statistics.

For the initial hydrocarbon detection (Supplementary Figure S1) and the temperature profiling (Figure 2), the hydrocarbon composition of *Synechocystis* WT and Δ*ado* was determined from unfractionated 1ml cell samples collected by centrifugation at 10min at ~16,000g (Sigma 1–14). The cell pellets were lysed by adding about an equal volume of acid washed glass beads (Sigma) and 800μl methanol followed by eight cycles of strong vortexing and cooling on ice. The samples were centrifuged for 5min at ~16,000g, and the supernatants were filtered through microcentrifuge tubes (Costar 8169 spin-X centrifuge filter 0.22μm) and transferred into GC–MS vials (Agilent) for analysis.

### Preparation of *Synechocystis* Membrane Fraction and Soluble Fraction for Hydrocarbon GC–MS and Western Blot Analysis

**Figure 3:**
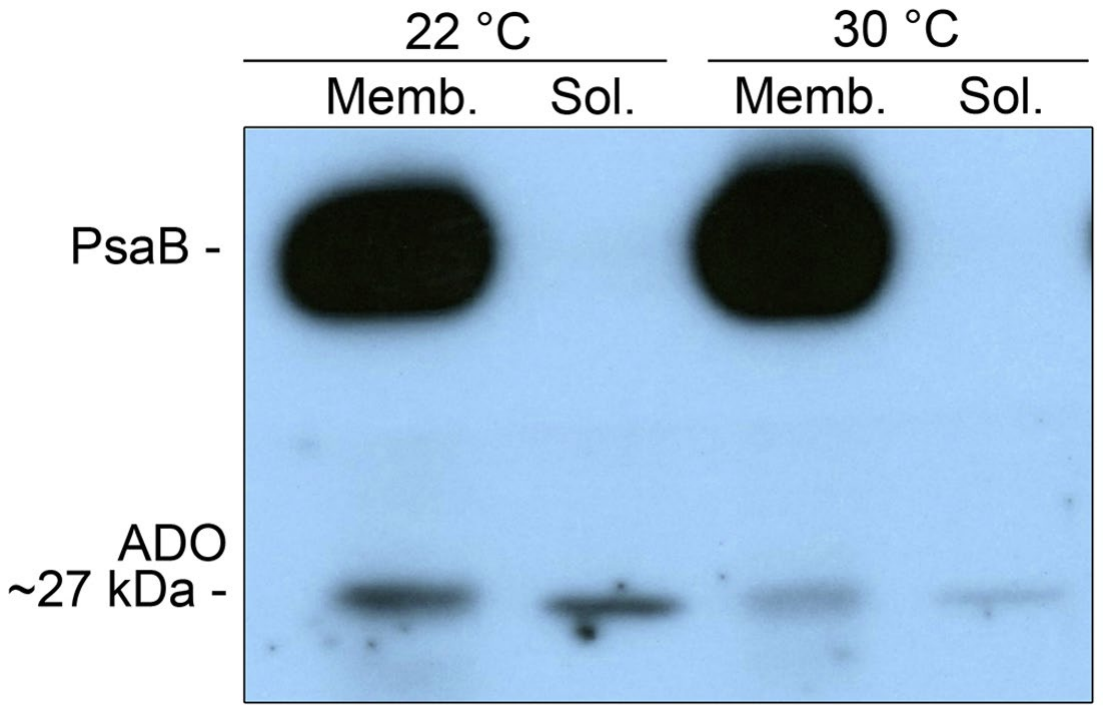
Western blot analysis of ADO in the membrane fraction (Memb.) and the soluble fraction (Sol.) of *Synechocystis* sp. PCC 6803 cells cultivated at 22°C and 30°C for 4days. The samples were loaded to the same final protein concentration (3μg) based on Bradford assay and analyzed using protein-specific antibodies against ADO, and the integral membrane reference protein PsaB. The analysis was carried out in four independent replicates.

For the cell fraction analysis (Supplementary Figure S2) and the Western blots (Figure 3; Supplementary Figures S6, S7), the samples were harvested form 25ml culture aliquots and suspended in 300μl of resuspension buffer [50mM Hepes-NaOH pH 7.5, 30mM CaCl_2_, and 800mM Sorbitol, 1mM Ɛ-amino-n-caproic-acid with Protease Inhibitor Mini Tablets (Pierce)]. After adding approximately 250mg of Zirconium oxide Beads 0.15mm (NEXT ADVANCE), the cells were broken with Bullet Blender^®^ Storm 24 (NEXT ADVANCE) to generate the *total cell extracts*. To separate *membrane fraction* from *soluble fraction*, 150μl of the total cell extracts was transferred into new microcentrifuge tubes and centrifuged for 40min at 16000g (Sigma 1–14). The *soluble fraction* was stored for analysis, and the *membrane fraction* was gently resuspended to 100μl of storage buffer (50mM Hepes-NaOH pH 7.5, 30mM CaCl_2_, 600mM sorbitol, 1 M betaine monohydrate with Protease Inhibitor Mini Tablets). Protein content of the cell samples was determined with the Bradford dye-binding method (Bradford, 1976) against bovine serum albumin standard from 0 to 25μg/ml. For the GC–MS analysis of hydrocarbons, the extracted samples (including a control sample from the culture medium) were mixed with methanol (HiPerSolv CHROMANORM, VWR Chemicals), centrifuged for 2min at ~16,000g at room temperature, and transferred into GC–MS vials (Agilent).

### Quantitative GC–MS Analysis of Hydrocarbons

Hydrocarbons were analyzed with Agilent 7890C gas chromatography equipment with an autosampler and a 5975C inert mass spectrophotometer (Agilent), using Innowax capillary column (260°C, 30m×0.32mm×0.5μm). Three microliters of the extracted culture sample was injected in a splitless mode (1.9mlmin^−1^ He flow, injector at 250°C), and the target hydrocarbons were separated using a temperature program: 55°C for 2min, then to 155°C with 20°C/min; 155–180°C with 8°C/min; 180–250°C with 20°C/min; finally 250°C for 10-min hold. The sample peaks were identified against commercial analytical standards (Sigma-Aldrich). Quantification was done by comparing the filtered MS peak areas (*m*/*z*=57 for C17 alkane; *m*/*z*=55 for C17:1 alkene and C15 alkane; *m*/*z*=83 for C15:1 alkene) to the corresponding standard peak signals.

### Generating ADO-Specific Antibody

In order to produce a specific antibody against ADO, the *ado* gene (*sll0208*) with additional 6x His-tag at the N-terminal end was amplified from genomic DNA and subcloned into a commercial pACYC plasmid (Novagen). The pACYC-sll0208 construct was confirmed by sequencing (MWG) and used for protein over-expression in *E. coli* BL21. Protein production was induced with 0.5mM isopropyl-β-d-thiogalactopyranoside (IPTG), and after ~16h, the cells were lysed with Cell Disrupter (Constant Systems Ltd) using 20 kpsi and the cell debris was removed. To purify the His-tagged ADO, the supernatant was mixed with 1ml pretreated TALON metal affinity resin and the bound protein was eluted with 20mM sodium phosphate pH 7, 100mM NaCl, 250mM imidazole, and 10% glycerol. The elution fraction with highest protein concentration was identified with Bradford reagent, and the target protein of ADO corresponding ~27kDa was separated with SDS-PAGE (Mini-PROTEAN^®^ TGX^™^, Sigma) and sent to Agrisera to raise a polyclonal antibody against ADO. The function of the antibody was verified in Western blot using different concentrations of fractionated and unfractionated cell samples.

### Western Blot Analysis

The samples for Western blot analysis were first subjected to SDS-PAGE using 12% acrylamide gel with 6M urea to separate proteins according to size. The samples were loaded based on the Bradford assay to 1–4μg protein per well (see each experiment for details), with 1:1 vol buffer containing 138mM Tris–HCl pH 6.8, 6M Urea, 22.2% glycerol, 4.3% SDS, 10% β-mercaptoethanol, and a small volume of Bromophenol blue. The separated proteins were electro-transferred from the acrylamide gel to Immobilon PVDF membrane (Millipore). The PVDF membrane was blocked with 5% milk (BIO-RAD blotting grade blocker) and incubated with first antibody and second antibody and finally with ECL^™^ Western blotting Detection Reagent (Amersham^™^ GE Healthcare) before imaging on film. The protein-specific antibodies used in the assay were α-ADO (custom made, see *Generating ADO-specific antibody* above) at 1:1000 dilution, α-PsaB (AS10 695, Agrisera) at 1:1000 dilution, and α-ATPase β (AS05 085, Agrisera) at 1:5000 dilution. The secondary antibody (AS09 602, HRP conjugated Goat anti-Rabbit IgG (H&L), Agrisera) was used at 1:10000 dilution.

### Microscopic Evaluation

For microscopic evaluation of cell number, size, and morphology, the *Synechocystis* strains were examined on a 0.1mm Bürker cuvette under a light microscope (Leitz Orthoplan Large Field Research Microscope) and photographed with a digital microscope camera (Leica DFC420C). The cells were visualized with Leica Application Suite V 4.1, and the horizontal diameter of 200 cells was measured in each case from the digital captures (in arbitrary units) using Adobe Photoshop CS4 Ruler Tool.

### Dry Cell Weight Analysis

In order to compare the dry cell weight (DCW) of WT *Synechocystis* and Δ*ado*, 5ml of cultures adjusted to same optical density at 750nm (OD_750_=1; Thermo Scientific Genesys 10S UV–Vis Spectrophotometer) was filtered through dried and pre-weighted microfiber membranes (VWR). After oven-drying at 98°C, the membranes were reweighted using an analytical scale (Sartorius MC1 Research RC 210P).

### Sedimentation Assay

To compare the sedimentation efficiency between the *Synechocystis* WT and Δ*ado* cells, the strains were grown at 30°C and atmospheric CO_2_ and adjusted to OD_750_=2.0 and 2.5. Sedimentation was monitored for 4days at RT in stationary 15-ml polypropene tubes, and pictures were taken at one-day intervals. After complete sedimentation (day 8), the resistance of the cell pellets toward resuspension was compared by shaking and vortexing the tubes.

### Quantifying Glycogen and Poly-β-Hydroxybutyrate

Commercial analytical kits were used for the quantitation of intracellular glycogen (Total Starch Assay Kit; Megazyme, United States) and intracellular poly-β-hydroxybutyrate (PHB; D-3-Hydroxybutyric Acid Assay Kit; Megazyme, United States) according to the manufacturers’ instructions and Thiel et al. (2017). To avoid the use of *Chl a* for normalization, the values were calculated as g per cell, by quantitating glycogen and PHB per ml culture, divided by the number of cells per ml culture.

### Pigment Analysis With Absorption Spectroscopy

Chlorophyll *a* (Chl *a*) content of the *Synechocystis* WT and hydrocarbon-deficient strain was determined by methanol extraction and spectrophotometric analysis at 665nm. Absorbance spectra from 400nm to 750nm was recorded on 96-well plates using a microplate reader (TECAN infinite M200 PRO) from 150μl culture samples adjusted to OD_750_ =0.25. Detailed pigment analysis was carried out with OLIS CLARiTY 17 UV/Vis spectrophotometer equipped with 8ml ICAM cuvette (On Line Instrument Systems, Inc., United States). For the measurement, the OD_750_ was adjusted to 0.3, and the obtained raw absorption values (AU) of the scans (370nm−700nm) were converted to real absorbance values (AU/cm) using Fry’s method (Fry et al., 2010).

### Quantification of Cellular Gas Fluxes

Membrane Inlet Mass Spectrometry (MIMS) was used to measure the steady-state cellular gas flux rates (O_2_ and CO_2_) directly from cell suspensions under increasing halogen light intensities of 0, 25, 50, 75, 100, 200, and 500μmol photons m^−2^ s^−1^ as previously described (Thiel et al., 2019). The cell samples were concentrated to Chl *a*=15μgml^−1^ and supplemented with 1mM total HCO_3_^−^ and 50μgml^−1^ carbonic anhydrase (bovine, Sigma). Gross O_2_ evolution rates were calculated from the addition of ^18^O_2_ gas (98%, CK Isotopes) based on equations and methods of (Beckmann et al., 2009).

### Fluorescence Measurements and P700 Absorbance

Chl fluorescence and P700 signals were recorded simultaneously with a pulse amplitude modulated fluorometer Dual-PAM-100 (Walz, Germany) from intact cells supplemented with 1mM ${\mathrm{HCO}}_{3}^{-}$ . The cell suspensions were adjusted to Chl *a*=15μgml^−1^, dark-acclimated for 10min, and treated with saturating pulses of 5,000μmol photons m^−2^ s^−1^ (300ms) and a strong far-red (FR) light (720nm, 75Wm^−2^). Applied actinic light was red light (620nm) of either 20μmol photons m^−2^ s^−1^ or 50μmol photons m^−2^ s^−1^. The light curves were recorded with 60-s illumination periods of gradually increasing actinic light intensities for samples without dark acclimation. The effective yield of PS II, [Y(II)], was calculated as (F_m′_-F_s_)/F_m′_ and of PSI, [Y(I)], as (P_m′_ – P)/P_m_.

### State Transition Analysis

Kinetics of the state transitions were recorded with a Dual-PAM-100 fluorometer from cell suspensions adjusted to Chl *a*=15μgml^−1^ and supplemented with 1mM HCO_3_^−^. The samples were dark-acclimated for 10min and illuminated with blue actinic light (460nm, 44μmol photons m^−2^ s^−1^) to induce State I. After 135s, the blue actinic light was switched to red actinic light (620nm, 44μmol photons m^−2^ s^−1^) to induce transition from State 1 to State 2, and after 215s, the light was turned back to blue to induce State I. During illumination, saturating pulses (5,000μmol photons m^−2^ s^−1^, 300ms) were applied to monitor the maximal fluorescence in light (F_m′_) and to calculate the effective yield of PS II, [Y(II)], as (F_m′_-F_s_)/F_m′_.

## Results

### Heptadecane and Heptadecene as the Native Hydrocarbon Markers in *Synechocystis*

In order to select specific native alkanes and alkenes for the target profiling, *Synechocystis* cell extracts were subjected to GC–MS analysis against commercial standards for the ions m/z 55, m/z 57, and *m*/*z*=83 to identify C15 and C17 hydrocarbon species typically found in cyanobacteria (Tan et al., 2011; Coates et al., 2014; Supplementary Figure S1). Besides several unidentified peaks, all the samples contained both C17 heptadecane and heptadecene (Supplementary Figure S1A), which were chosen for the subsequent trials as the representatives of saturated alkanes (derived from *de novo* biosynthesis or *via* membranes) and unsaturated alkenes (derived only *via* membranes), respectively (see Figure 1). These target compounds were found exclusively in the insoluble fractions, and none were detected in the supernatant or the growth medium (Supplementary Figure S2). In contrast, C15 pentadecane and pentadecene were not present at detectable concentrations in any of the samples (Supplementary Figure S1B).

### Temperature and Osmotic Pressure as Changing Variables for Cell Cultures

Four different cultivation temperatures (15°C, 22°C, 30°C, and 38°C) and osmotic pressure (0.5M supplied sorbitol) were selected as the conditional variables for the analysis of *Synechocystis* hydrocarbon production, based on earlier observed responses of ADO or AAR expression under similar conditions (Singh et al., 2010). In the absence of sorbitol, the clearest temperature-specific differences in cell propagation were recorded during the first 4days of the cultures (Supplementary Figure S3), and while the cell densities under 15°C, 22°C, and 30°C reached similar levels by the day 12 (Supplementary Figures S3A–C), the overall growth was clearly suppressed at 38°C (Supplementary Figure S3D). The pigment profiles remained relatively similar between the lower temperatures (Supplementary Figures S4A–C), while the relative content was lower in cells cultured at 38°C (Supplementary Figure S4D). Additional supplementation of sorbitol systematically reduced growth at 15°C (Supplementary Figure S3A), 30°C (Supplementary Figure S3C), and 38°C (Supplementary Figure S3D), but interestingly, not at 22°C (Supplementary Figure S3B). Sorbitol also altered the relative amounts of carotenoids, phycobilisomes, and Chl *a* between the cultures (Supplementary Figures S4A–C).

### Hydrocarbon Production Patterns Respond to Changes in Temperature

Heptadecane and heptadecene were quantified at five successive time points (days 0, 1, 2, 4, and 8) from *Synechocystis* cells cultured under the eight selected conditions (15°C, 22°C, 30°C, and 38°C, with and without sorbitol; Figure 2). Despite the variation between the very first sampling points, which expectedly reflected the transition from the common 30°C preculture conditions to each target temperature, it was clear that the overall quantitative and qualitative hydrocarbon composition of the cells was significantly affected by the cultivation temperature (Figures 2A–D). The intracellular heptadecene content was dramatically reduced in response to the increase in temperature, as seen in gradual decline from 15°C (Figure 2A) toward 38°C (Figure 2D). In contrast, the overall heptadecane levels were lowest at 15°C (Figure 2A) and maintained constantly high throughout the higher temperature range (Figures 2B–D). Statistical comparison between the cultivation temperatures and the hydrocarbon levels recorded on day 8 (Supplementary Figure S5, Table S2) clearly confirmed these trends, showing the decrease in the amount of unsaturated heptadecene (Supplementary Figures S5A,B) and the corresponding increase in the saturated heptadecane (Supplementary Figures S5C,D) upon shift toward higher temperatures, and the inverse correlation between the two products (Supplementary Figures S5E,F). Unlike the temperature, increase in hypertonicity resulting from sorbitol supplementation (Figure 2; Supplementary Figure S5) had only minor effect on the hydrocarbon production profiles.

### ADO Is Partially Associated With Membranes and Expressed Differentially Under Varying Growth Conditions

To study the expression patterns of ADO in *Synechocystis*, a polyclonal antibody was generated against the protein (Supplementary Figure S6) and used for Western blot analysis. The cultivation temperatures 22 and 30°C were selected for the comparison because of the clear differences observed between the corresponding WT hydrocarbon profiles (Figures 2B,C), while the overall growth (Supplementary Figures S3B,C,C) and absorbance properties (Supplementary Figures S4B,C) remained largely unaffected. Although ADO has no integral membrane domains and is known to be exclusively expressed in soluble form, the enzyme was detected in both the soluble fraction and the insoluble membrane fraction (Figure 3). The analysis also revealed that ADO was expressed at higher levels at 22°C in comparison with 30°C, as seen in both the fractionated (Figure 3) and unfractionated (Supplementary Figure S7) samples. Additional supplementation of sorbitol appeared to have a slight negative impact on the expression of ADO under both temperatures (Supplementary Figure S7), although statistical difference could not be verified due to total intensity variations between the replicates.

### Inactivation of ADO Changes the Growth and Optical Properties of the Cells

In order to study the effects associated with the lack of hydrocarbons in the cell, the gene encoding for ADO (*sll0208*) was inactivated by inserting a kanamycin resistance cassette in the middle of the coding region by homologous recombination. The resulting *Synechocystis* strain *ado*::KmR (Δ*ado*) was confirmed segregated (Supplementary Figure S8) and, as expected, had lost the capacity to produce alkanes and alkenes (Supplementary Figure S1C). The phenotype was characterized by comparing the Δ*ado* strain against the WT in respect to the final OD_750_, cell count, DCW, cell size, and Chl *a* content after a four-day cultivation under 50μmol photons m^−2^ s^−1^ of constant light at 22°C and 30°C (Table 1). Based on the recorded OD_750_ change and the corresponding number of cells, the Δ*ado* strain appeared to grow slower than the WT under both temperatures (Table 1 and Supplementary Figure S9). However, the Δ*ado* cells were systematically larger in size, which is likely to affect the optical properties and potentially the spectrophotometric comparison between the strains (Malerba et al., 2018; Table 1). Accordingly, the Δ*ado* strain was shown to have more biomass than the corresponding WT culture based on DCW, despite the lower OD_750_ value at the end of the four-day cultivation period at 30°C (Table 1). On the contrary, when cultured at 22°C, the Δ*ado* strain was shown to have lower DCW as compared to the WT control, which was consistent with the reduced growth based on OD_750_ (Table 1). Comparison of the strains in respect to the Chl *a* content, measured per culture volume on cultivation day 4, showed significantly lower relative chlorophyll level in the Δ*ado* strain both at 22°C and at 30°C (Table 1). This difference was more pronounced than the corresponding changes observed in either cell size or biomass formation, implicating that the absolute Chl *a* per cell and per biomass was reduced in response to the disruption of *ado*. This has a direct impact on all the subsequent comparative analyses between Δ*ado* and the WT strain that rely on chlorophyll normalization and had to be taken into account in result interpretation.

**Table 1:** Comparison of Δ*ado* and WT *Synechocystis* sp. PCC 6803 in respect to differences in optical density (OD_750_), chlorophyll *a* (Chl *a*) content, cell number, dry cell weight (DCW) and diameter of the cell after growing the cells for 4days at 22°C and 30°C (starting from OD_750_ 0.5).

Strain	OD	%	Chl α	%	(cells L^−1^) Cell count	%	(gL^−1^) DCW	%	(a.u.) Diameter	%	(a.u.) Cell volume	%
WT 22°C	1.22 ± 0.0436	100	7.76 ± 0.513	100	3.77^*^10^12^ ± 0.540×10^12^	100	0.324 ± 0.0269	100	0.28 ± 0.0120	100	1.15×10^−2^	100
Δ*ado* 22°C	0.95 ± 0.0486^*^	78	4.50 ± 0.426^*^	58	3.07×10^12^±0.515×10^12^	81	0.281 ± 0.0188	87	0.31 ± 0.0170^*^	111	1.56×10^−2*^	136
WT 30°C	1.23 ± 0.0798	100	8.57 ± 0.365	100	4.36×10^12^ ± 0.974×10^12^	100	0.285 ± 0.0233	100	0.28 ± 0.0140	100	1.15×10^−2^	100
Δ*ado* 30°C	1.11 ± 0.0381^*^	90	5.73 ± 0.00970^*^	67	3.87×10^12^ ± 0.588×10^12^	89	0.332 ± 00311^*^	116	0.32 ± 0.0170^*^	114	1.72×10^−2*^	150

*The cells were assumed to be spherical, and the corresponding cell volumes were calculated from the diameters using the formula V=4/3πr^3^. The averages and standard deviations were calculated based on three independent replicates. Asterisks indicate statistically significant (p<0.05) differences in the measured parameters in the Δado mutant strain in comparison to WT under 22°C and 30°C, as analyzed using two-sided two-sample t test*.

### Δ*ado* Cells Sediment Faster in Comparison to the WT

**Figure 4:**
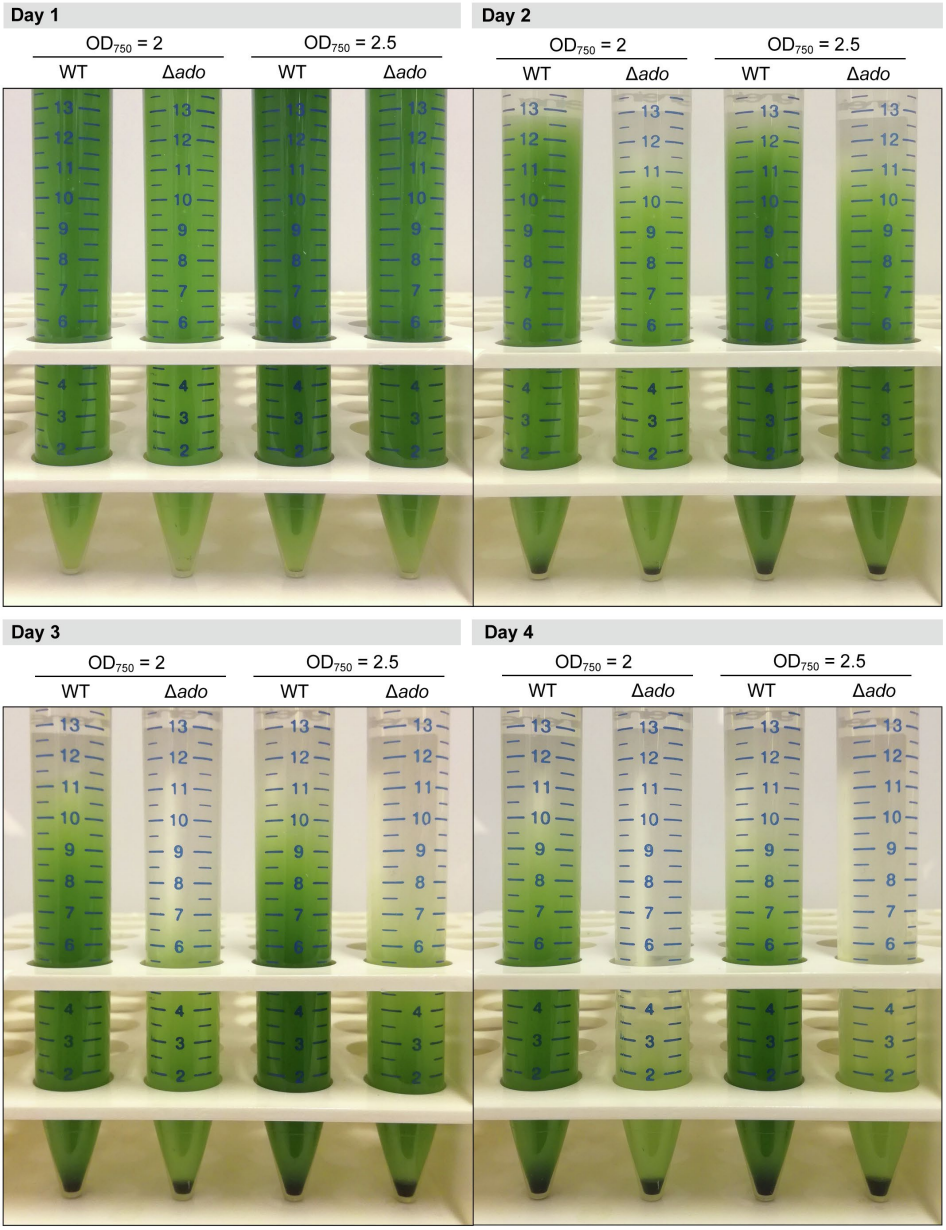
Comparison of static gravitational sedimentation between *Synechocystis* sp. PCC 6803 Δ*ado* and WT strains at optical cell density (OD_750_) 2.0 and 2.5. The pictures were taken on subsequent days 1–4. For the experiment, the cells were cultivated at 30°C and atmospheric CO_2_.

During cultivation and handling of liquid suspensions, the Δ*ado* cells were observed always to sediment faster than the WT cells. As visualized in BG11 cultures left standing still in test tubes at room temperature, majority of the Δ*ado* cells settled to the bottom by the end of the four-day incubation (Figure 4), while for the WT cells, it took over twice the time. In parallel, the Δ*ado* strain formed clearly more compact durable pellets than the WT, as demonstrated by the resistance toward resuspension by vigorous shaking and vortexing after complete sedimentation (Supplementary Figure S10).

### The Δ*ado* Cells Accumulate Increased Amounts of Glycogen and PHB

To assess differences in carbon allocation toward storage compounds and potential consequent effects on cell density, the Δ*ado* and WT strains were compared in respect to intracellular glycogen and PHB content. When calculated as weight per cell, the Δ*ado* strain was shown to accumulate more glycogen than WT both at 22°C (481%) and at 30°C (335%; Table 2). This increase was clearly more profound than the corresponding change in the cell size (Table 1), implicating that the inactivation of the *ado* was systematically accompanied by glycogen storage build-up. There was also a slight increase in PHB content in the Δ*ado* strain (132% at 22°C and 166% at 30°C; Table 2), but this roughly correlated with the changes observed in the cell size (Tables 1 and 2).

**Table 2:** Quantification of storage compounds glycogen and poly-β-hydroxybutyrate (PHB), per cell from Δ*ado* and WT *Synechocystis* sp. PCC 6803 cultures grown for 4days at 22°C and 30°C.

Strain	(g per cell) Glycogen	%	(g per cell) PHB	%
WT 22°C	4.32×10^−16^ ± 3.21×10^−16^	100	2.00×10^−16^ ± 0.901×10^−16^	100
Δ*ado* 22°C	20.8×10^−16^ ± 13.6×10^−16^	481	2.63×10^−16^ ± 0.669×10^−16^	132
WT 30°C	5.02×10^−16^ ± 1.97×10^−16^	100	1.46×10^−16^ ± 0.368×10^−16^	100
Δ*ado* 30°C	16.8×10^−16^ ± 3.27×10^−16*^	335	2.43×10^−16^ ± 0.433×10^−16*^	166

*Glycogen was broken down and quantified as glucose, and the averages and standard deviations for both compounds were calculated based on three biological replicates. Asterisks indicate statistically significant (p<0.05) differences in the measured parameters in the Δado mutant strain in comparison to WT under 22°C and 30°C, as analyzed using two-sided two-sample t test*.

### Inactivation of Hydrocarbon Biosynthesis Results in Altered Pigment Profile

**Figure 5:**
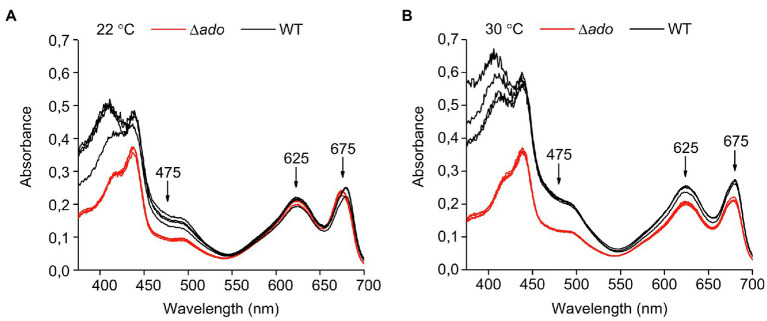
Absorption profiles of *Synechocystis* sp. PCC 6803 WT (black line) and Δ*ado* (red line) strains recorded between 370–700nm from four independent replicates. The cells were grown for 4days at **(A)** 22°C and **(B)** 30°C and adjusted to OD_750_=0.3 before the measurement. The arrows indicate the absorption maxima for carotenoids (~475nm), Chl *a* (~625nm) and phycocyanin (~675nm).

In order to evaluate changes in the cellular pigment content resulting from the lack of native hydrocarbons, the absorbance profiles (370–700nm) of the Δ*ado* and WT strains were compared between cultures grown at 22°C (Figure 5A) and at 30°C (Figure 5B). Although unambiguous quantitative comparison of individual pigments was difficult due to the variation in the normalization parameters (see above), the results showed that the relative amount of carotenoids (~475nm) in comparison with both Chl *a* (~625nm) and phycocyanin (~675nm) was reduced in the Δ*ado* strain, especially in cells cultured at 22°C.

### Total Carbon Uptake and Oxygen Evolution Capacity Are Reduced in the Δ*ado* Strain

**Figure 6:**
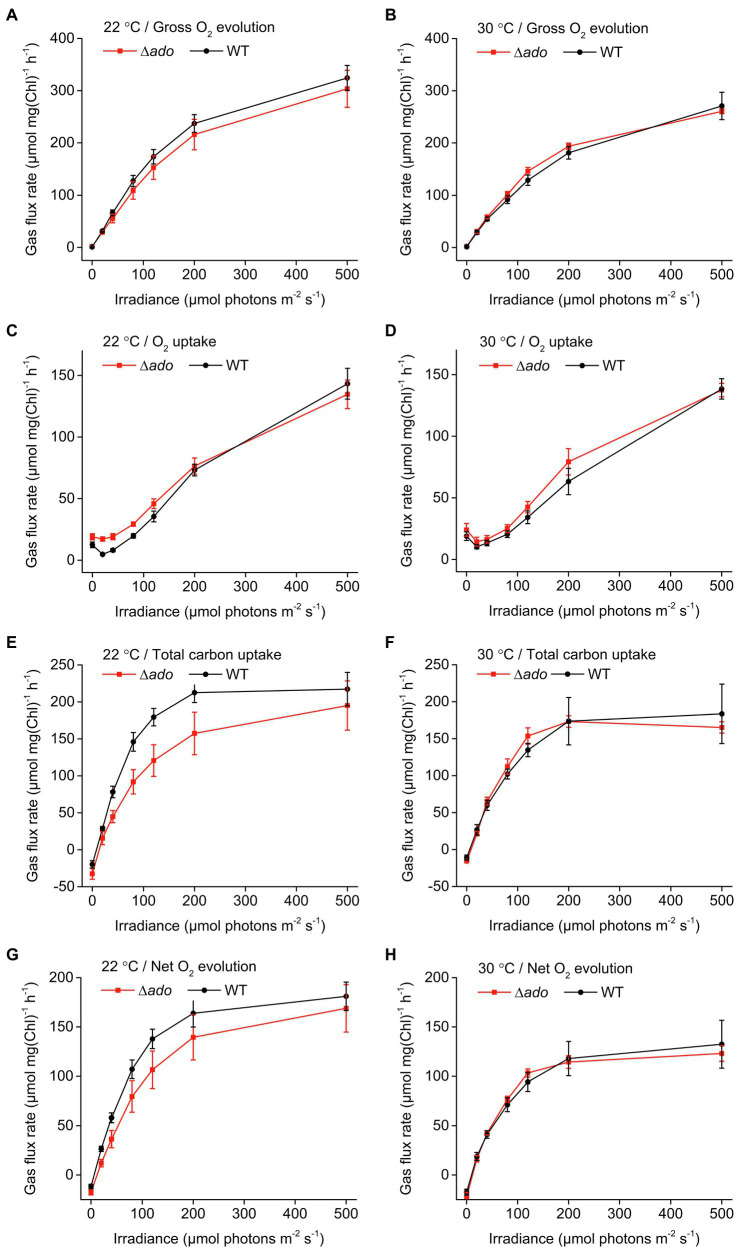
Comparison of cellular gas fluxes between *Synechocystis* sp. PCC 6803 WT and *Δado* measured by Membrane Inlet Mass Spectrometry (MIMS): **(A,B)** gross O_2_ evolution, **(C,D)** O_2_ uptake, **(E,F)** total carbon uptake, and **(G,H)** net O_2_ evolution. The cells were cultivated for 4days at 22°C or at 30°C as indicated, and each sample was adjusted to Chl *a*=15μgml^−1^ for analysis. The averages and standard deviations were calculated based on three independently conducted trials.

To assess changes in photosynthetic gas fluxes resulting from the inactivation of hydrocarbon biosynthesis in *Synechocystis*, the Δ*ado* and WT strains were subjected to MIMS analysis (Figure 6). The results revealed a systematic slight reduction in practically all the measured parameters in the Δ*ado* cells grown at 22°C, including gross oxygen evolution (Figure 6A), net O_2_ evolution (Figure 6G) and uptake (Figure 6C), and total carbon uptake (Figure 6E). Taking into account the lower Chl *a* content of the Δ*ado* strain (Table 1), these differences were even more distinct when compared to WT per cell or per biomass. For the same reason, the corresponding gas fluxes measured for the cells grown at 30°C, which appeared to remain mostly unchanged (Figures 6B,D,F,H), were in fact also lower in the Δ*ado* cells. This implied that the photosynthetic activity of the *Synechocystis* Δ*ado* mutant was systematically impaired in comparison with the WT strain on the basis gross oxygen evolution (which is a direct measure of PSII activity) and the capacity to fix carbon under both conditions tested.

### Fluorescence Analysis Reveals Minor Changes Only in the Function of PSII

In order to evaluate the state of the individual photosystem complexes in the cells impaired in hydrocarbon biosynthesis, the Δ*ado*, and WT strains grown at 22°C and 30°C were subjected to detailed chlorophyll fluorescence analysis using a pulse amplitude modulated fluorometer Dual-PAM-100 (Supplementary Figure S11). As all the calculated parameters are relative functional values that are not dependent on the absolute chlorophyll amount, the difference in the Chl *a* content of the strains (Table 1) was not expected to affect the functional comparison of the samples. Generally, there were no dramatic differences between the Δ*ado* and WT strains, and the variations observed in the individual fluorescent parameters seemed to be relatively insignificant in regard to the overall system performance (Supplementary Figure S11). The Δ*ado* PSII yield [Y(II)] was consistently slightly higher than in the WT and apparent especially under lower actinic light intensities (Supplementary Figures S11E vs. I, Q vs. U). In accordance, there were no signs of donor side limitation [Y(ND)], supporting the view that the electron flux from PSII was not restricting the system capacity in either of the strains (Supplementary Figures S11F,J,R,V). In regard to the PSI parameters, there were no marked differences between the strains: The Δ*ado* PSI yield [Y(I)] was practically unchanged (Supplementary Figures S11C,O), while the recorded decrease in acceptor side limitation [Y(NA)] seemed insignificant (note the axis scale; Supplementary Figures S11L,T,X). The cultivation temperature, 22°C (Supplementary Figures S11A–L) versus 30°C (Supplementary Figures S11M–X), or actinic light intensity from 20 to 350μmol photons m^−2^ s^−1^ (Supplementary Figures S11A–D vs. M–P) seemed not to be relevant in respect to the monitored parameters in either of the strains.

### Movement of Phycobilisomes Appears to Be Restricted in the Δ*ado* Strain During State Transitions

To further evaluate the relatively high PS II capacity of the Δ*ado* strain, the cells were subjected to induced state transition analysis, where the movement of phycobilisomes from PS I (state 2) to PS II (state 1) and back was induced by changes in actinic light quality (blue – red – blue; Supplementary Figure S12). In the Δ*ado* strain Y(II) measured from dark-adapted cells prior any illumination was higher than in WT (first measuring point in Supplementary Figures S11E,I,Q,U, S12) without changes in the F_0_-level (Supplementary Figure S12), suggesting that larger proportion of phycobilisomes was associated with PSII than with PSI in the dark. In the absence of light, the phycobilisomes in cyanobacteria are generally more extensively coupled to PSI than PSII which is likely caused by the reduced plastoquinone (PQ) pool as a result of respiration (Mullineaux and Allen, 1990). In addition, the movement of phycobilisomes was only moderate upon the transition from blue to red actinic light (from state 2 to state 1) and back in the Δ*ado* strain compared to WT (Supplementary Figure S12). Although the limited movement of the phycobilisomes in the Δ*ado* strain during state transitions was obvious at both temperatures, the difference was more profound at 22°C (Supplementary Figure S12A) than at 30°C (Supplementary Figure S12B).

## Discussion

### Evaluating the Biological Role of Cyanobacterial Hydrocarbon Saturation

Cyanobacteria are exceptional prokaryotes due to their ability to perform oxygenic photosynthesis. This is linked to many unique structural and functional features that are not found elsewhere in the bacterial kingdom, one of which is the conserved universal capacity to produce aliphatic hydrocarbons. The hydrocarbons are typically medium chain length alkanes and alkenes (Coates et al., 2014) found both in the cyanobacterial plasma membrane and the thylakoid membrane (Lea-Smith et al., 2016), where they are expected to affect the physicochemical properties of the lipid bilayers and associated components – including the photosynthetic machinery. Interestingly, no single key function has been assigned for the hydrocarbons which would be essential for the survival of cyanobacteria, although they have been shown to be necessary for optimal photoautotrophic performance and other (possibly indirect) metabolic functions related to the acclimation to natural environments. The current study expands our perception of the biological role of aliphatic hydrocarbons in cyanobacteria and provides new information specifically on the relative content between alkanes and alkenes under varying temperatures and osmotic pressure in *Synechocystis*. Instead of looking at intracellular hydrocarbons as a bulk of nonpolar hydrophobic carbon chains, understanding the mechanisms that modulate the ratio between the saturated and unsaturated species, and the synchronization of different enzyme components involved, helps us explain many of the changes that occur upon inactivation of hydrocarbon biosynthesis.

### Membrane Composition Is Adjusted by Reducing Hydrocarbon Saturation Under Lowered Growth Temperatures

The hydrocarbon products quantified in *Synechocystis* in this study were C17 heptadecane (synthesized *de novo* or derived from membranes) and heptadecene (derived exclusively from membrane-associated precursors; see Figure 1), which were used for monitoring changes in the carbon chain saturation. Our key finding was that the relative ratio between heptadecane and heptadecene varied significantly according to the cultivation temperature. The relative abundance of these compounds was measured over an eight-day time course at four different temperatures, which univocally demonstrated that the level of saturation decreases upon reduced temperatures and increases at elevated temperatures (Figure 2; Supplementary Figure S5, Table S2), while the total hydrocarbon content is not dramatically altered (Supplementary Figures S2, S7). These changes followed the same trend as generally known to occur in membrane lipids, where the acyl chain saturation is adjusted by increasing the number of double bonds in response to lowered temperatures (Wada et al., 1994; Inoue et al., 2001; Mikami and Murata, 2003; Los and Murata, 2004; Los et al., 2013). This sterically hinders the stacking of the lipid molecules and results in increased membrane fluidity [see review (Mikami and Murata, 2003)], which is generally essential for the lateral movement, and the interactions and function of integral proteins. Corresponding temperature-dependent regulation of hydrocarbon saturation has been reported between 1-nonadecene and 1,14-nonadecadiene in the alternative Ols pathway in *Synechococcus* 7002 (Mendez-Perez et al., 2014), which supports the idea of a similar response mechanism in different cyanobacterial species, irrespective of the pathway in question. Notably, to our knowledge, polyunsaturated alkenes have not been reported in *Synechocystis*, despite the presence of 18:2, 18:3, and 18:4 fatty acyl precursors, that are the key components in the membrane fluidity response (Wada and Murata, 1990). This could, at least in part, reflect restrictions in the substrate specificity of the downstream enzymes, as in the case of ADO, which has a fairly narrow ligand-binding channel that may not readily accommodate bulky carbon chains with multiple double bonds (Buer et al., 2014; Bao et al., 2016).

In the ADO/AAR pathway in *Synechocystis*, the production appears to be regulated at several levels that together dictate the amount, the carbon chain length, and the saturation of the resulting hydrocarbons. As the acyl chain double bond formation is brought about by the membrane-associated desaturases, the availability of desaturated ACP-linked substrates for AAR/ADO is dependent on the activity of (i) LipA that releases fatty acids from the membrane and (ii) Aas that attaches the precursors to ACP. At the next level, the substrate specificity of (iii) AAR determines which precursors are released form ACP and become accessible for (iv) ADO, hence defining the hydrocarbon profile in a stepwise manner *in vivo*. In the case of the targets in this study, the exclusive presence of heptadecane and heptadecene (Supplementary Figure S1A) indicates that AAR in *Synechocystis* catalyzes primarily the conversion of the C18 intermediates, while the C16 precursors remain unreacted, as seen in the absence of pentadecane and pentadecene (Supplementary Figure S1B). This is in agreement with earlier reports showing that AAR substrate preference is higher toward C18 substrates in freshwater species such as *Synechocystis*, as opposed to preference for C16 substrates in marine species (Kudo et al., 2016). Linking these observations back to the cellular lipids, the C18 precursors in *Synechocystis* originate specifically from the sn-1 position of the glycerol moiety, where the first carbon chain desaturation always takes place at position ∆^9^ (Wada and Murata, 1990) by the action of DesC [see review (Los and Murata, 2004)]. The resulting n-9 monounsaturated fatty acyl precursor is consistent with the formation of 8-heptadecene *via* the AAR/ADO pathway, which is the predominant desaturated hydrocarbon identified in *Synechocystis* (Tan et al., 2011; Lea-Smith et al., 2015). Intriguingly, however, unlike the following desaturase reactions (DesA; ∆^12^, DesB; ∆^15^, DesD¸∆^6^), the initial ∆^9^ DesC desaturation is not temperature-regulated in *Synechocystis* (Wada and Murata, 1990; Los et al., 1997). This clearly implicates that the observed increase in the amount of heptadecene at lower temperatures is not controlled at the level of the desaturases, unlike in the case of the membrane lipids. Therefore, it is still unclear how the hydrocarbon temperature response is synchronized, but based on the findings, it must involve regulated enzyme-catalyzed steps downstream DesC.

At the last step of hydrocarbon biosynthesis, ADO catalyses the cleavage of C18 aldehyde into the corresponding C17 hydrocarbon product and formate. Our results suggest that the overall activity may be maintained relatively constant despite the changes in cultivation conditions: The expression of ADO is enhanced upon the shift from 30 to 22°C (Supplementary Figure S7), which may be a way to compensate for the expected reduction in catalytic performance at reduced temperatures. This implies that hydrocarbon production would be regulated primarily through precursor availability, rather than the final ADO-catalyzed step, in agreement with relatively constitutive expression patterns of ADO reported earlier (Klähn et al., 2014). From the viewpoint of strain engineering aiming to overproduce hydrocarbons in cyanobacteria, increase in the intracellular concentration of ADO could therefore have only a limited effect on efficiency, unless coupled to concomitant increase in the local concentration of accessible substrates *via* the upstream steps. Although the amount of hydrocarbons in *Synechocystis* has been reported to change in response to temperature shifts (Berla et al., 2015), our results strongly suggest that changes in the saturation of the compounds may be more relevant in regards to the biological function than earlier anticipated and sometimes more dramatic than the alterations in the total concentration.

In a similar manner to decreased temperatures, certain cyanobacteria (Huflejt et al., 1990) and other microorganisms (López et al., 2000) have been reported to respond to osmotic stress by increasing the desaturation of membrane lipids. Although the molecular interactions are not comprehensively understood, in cyanobacteria this is appears to protect the photosynthetic machinery against damage upon exposure to high salinity [see review (Yang et al., 2020)]. However, the physiological effects and cellular response mechanisms are highly osmolyte-specific (Kanesaki et al., 2002; Marin et al., 2006) and depend on the organism in question. The composition of aliphatic hydrocarbons has also been shown to respond to increased salt concentrations in *Synechococcus elongatus* PCC 7942, *Aphanothece halophytica* (Yamamori et al., 2018) and *Anabaena cylindrica* (Bhadauriya et al., 2008), although the impact on the saturation profiles has not been studied. In the current work, we demonstrated that the increase in osmotic pressure by the supplementation of 0.5M sorbitol does not result in any notable changes in the total hydrocarbon content or saturation in *Synechocystis* (Figure 2; Supplementary Figures S5, S7, Table S2). This suggests that the AAR/ADO pathway is not involved in the regulation of sorbitol-induced osmotic stress, unlike observed for the temperature shifts.

### Membrane Association of ADO Is Consistent With the Localized Targeted Function of Hydrocarbons

The total amount of hydrocarbons in the cyanobacterial membranes is relatively low as compared to the lipids (Coates et al., 2014), and the function is expected to base on rather unspecific physicochemical interactions, such as hydrophobicity and molecular topology. Consequently, although found both in the plasma membrane and the thylakoid membrane (Lea-Smith et al., 2016), it appears unlikely that these compounds would be evenly distributed to induce uniform global effects like the lipid acyl chains that control the overall membrane system fluidity. Instead, we anticipate the hydrocarbons to have a more localized role at specific confined parts of the cell, particularly in the thylakoid membrane – in association with the photosynthetic machinery. *In silico* modeling supports molecular clustering of the hydrocarbon chains in between the lipid layers, which results in localized changes in acyl chain organization and molecular dynamics (Lea-Smith et al., 2016; Misuraca et al., 2021). Such architecture could suggest that the distribution of hydrocarbons is not random but controlled and may be determined by the physical localization of ADO and other enzymes to enable targeted production *in situ*. This is in agreement with our observation that ADO is always found also in the membrane fractions of the cell (Figure 3; Supplementary Figure S6), despite the fact that the enzyme is expressed in soluble form without identified transmembrane domains that would directly link to the thylakoid membrane. However, the mechanism for possible membrane association, and hence the placement of ADO in respect to other membrane components, is unclear. Besides electrostatic interactions with AAR that are important for the catalysis (Chang et al., 2020), ADO has been previously shown to interact with at least seven proteins in a yeast two-hybrid experiment (Sato et al., 2007). These include a group 2 RNA polymerase sigma factor SigD (encoded by *sll2012*), photosystem I assembly-related protein YCF39 (encoded by *slr0171*), 5-formyltetrahydrofolate cyclo-ligase (encoded by *sll1643*), and an essential cytokinetic protein FtsQ involved in cell division (encoded by *sll1632*; Marbouty et al., 2009), in addition to three unknown or hypothetical proteins (encoded by *sll1218*, *slr0243* and *slr1398*). According to UniProt database, the proteins encoded by *slr0171* and *sll1632* contain transmembrane domains and could thus function as direct docking partners for ADO on the membrane, although other interactions, direct or indirect mediated *via* other soluble proteins, cannot be ruled out.

### Growth Defects of ∆*ado* Strain Are Apparent Especially Under Lowered Temperatures

The inactivation of hydrocarbon biosynthesis in cyanobacteria has been previously shown to result in defected growth (Berla et al., 2015; Lea-Smith et al., 2016). This has been linked with changes in cell size and morphology (Lea-Smith et al., 2016), which has a direct impact on the optical properties and may complicate the comparison between strains, especially as the effects vary depending on environmental factors such as temperature (Berla et al., 2015). To determine the conditional effects on cell proliferation under the setup used in this work, the WT *Synechocystis* and ∆*ado* were analyzed in parallel for changes in OD_750_, Chl *a* content, DCW, and cell number and size (Table 1), as well as overall absorbance properties (Figure 5), for cultures grown at 30°C and 22°C. The comparison clearly confirms that (i) none of the parameters alone properly describe the growth of the cells and that (ii) the correlations may critically change upon shift in temperature, which has to be taken into account in all experiments that base on normalization on OD_750_ or Chl *a*. Importantly, the results indicate that the growth of the ADO deletion mutant at lowered culture temperature is defective based on all the measured parameters, OD_750_, cell number and DCW, while the cell size is systematically increased (Table 1). In context with our functional hypothesis, this is expectedly caused by the inability of the ∆*ado* cells to maintain optimal lipid bilayer dynamics under reduced temperatures, which is normally locally adjusted by altering the saturation of the membrane-embedded hydrocarbons. Our observations are well consistent with earlier reports on growth defects in hydrocarbon-deficient strains (Berla et al., 2015; Lea-Smith et al., 2016) and allow us to link the condition-dependent phenotypic changes (Table 1) further with changes in the hydrocarbon saturation (Figure 2), cellular gas fluxes (Figure 6), and biophysical parameters (Supplementary Figures S11, S12) as discussed below.

### Inability to Adjust Thylakoid Membrane Hydrocarbon Composition Under Reduced Temperature Results in Impaired Photosynthesis

It has now been established that hydrocarbons are constituents of the cyanobacterial thylakoid membranes and that their saturation level is regulated by temperature (Figure 2; Supplementary Figure S5, Table S2). Based on model membrane vesicles, the presence of hydrocarbon chains has been shown to significantly affect the molecular dynamics and organization of multilamellar membranes (Misuraca et al., 2021), while factors that induce lamellae segregation result in increased membrane fluidity, especially within the hydrophobic lipid bilayer interior (Nami et al., 2021). This could be a mechanism to locally control the lateral diffusion and functional interaction of the integral thylakoid proteins and associated components, that are directly linked with, for example, the efficiency of photosynthetic electron transfer and the PSII D1 repair cycle (Nami et al., 2021), and the rate of quinone pool oxidation (Maksimov et al., 2017). Therefore, by altering the saturation (i.e., the packing properties) of the hydrocarbon chains, the cells may be able to modulate the plasticity of specific parts of the thylakoid membrane in response to changing environments, such as decreased temperatures. In agreement, modeling studies have indicated that incorporation of hydrocarbons in the thylakoids increase the membrane thickness and induce local swelling by reducing lipid chain order and packing efficiency (Lea-Smith et al., 2016). This has been shown to be linked with the thylakoid membrane curvature in hydrocarbon-deficient *Synechococcus* sp. PCC 7002 ∆*Ols* strain (Lea-Smith et al., 2016), and expected to be accompanied by changes in the organization properties and further, on the fluidity of the membranes (Nami et al., 2021). In line with the hypothesis of temperature-related control, we observed several changes that appeared to be more profound under lowered temperatures, including reduced relative carotenoid content (Figure 5), increased glycogen accumulation (Table 2), impaired phycobilisome movement during state transitions (Supplementary Figure S12), and downregulation of gross O_2_ evolution of PSII (Figure 6A) and CO_2_ fixation (Figure 6E) in the ∆*ado* strain. We also observed slight upregulation of light-induced O_2_ uptake in the ∆*ado* mutant grown at 22°C (Figure 6C), which is in line with the proportional increase in cyclic electron transport (CET; Berla et al., 2015) and the associated oxygen consumption by the terminal oxidases [e.g., Cyd and Cox (Ermakova et al., 2016)]. However, the direct effects measured for on the PSII and PSI fluorescence parameters appear to be relatively insignificant (Supplementary Figure S11), as reported earlier (Lea-Smith et al., 2016), and without any clear temperature dependence. This apparent discrepancy between the overall effects and the individual steps through PSII to PSI suggests that the molecular level effects resulting from the absence of hydrocarbons may be small, but together sum up to prevent optimal fine-tuning of the system at lowered temperatures with consequent general metabolic-level changes.

### Inactivation of Hydrocarbon Biosynthesis Links to Altered Central Carbon Metabolism

The relative increase observed for CET is expected to increase of the intracellular ATP/NADPH ratio (Berla et al., 2015), which again is likely to have an effect on the carbon partitioning, and hence the metabolic flux distributions in the cell. In agreement, we observe dramatic increase in the glycogen accumulation in the ∆*ado* cells grown at reduced temperatures (Table 2), a phenomenon which is known to be tightly linked with high ATP levels over NADPH, as part of the mechanisms regulating the metabolic redox homeostasis in cyanobacteria (Saha et al., 2016; Huokko et al., 2017; Shinde et al., 2020). Besides the overall slight reduction in the photosynthetic efficiency, the build-up of glycogen reserves as an alternative for allocating the carbon efficiently for the biosynthesis of cellular building blocks, such as lipids and proteins, may also be a factor contributing to the reduced growth of the ∆*ado* mutant. In addition, the accumulation of the highly dense glycogen has a clear impact on the buoyancy of the hydrocarbon-deficient cells, which sink faster in solution than the corresponding WT cells (Figure 4; Supplementary Figure S10). As the hydrocarbons are unlikely to affect the flotation properties of the cells to any significant extent, due their relatively low total quantity in the native cells, this effect is likely to be merely an indirect artefact resulting from altered carbon metabolism of the ∆*ado* strain.

## Summary

The evolutionary conservation of cyanobacterial hydrocarbon biosynthesis in itself is a strong indication that these compounds must have an intrinsic biological role that provides a clear physiological advantage in nature. Despite the vast amounts of collected structural and functional molecular level information on the metabolic steps and characterized hydrocarbon-deficient mutants, we are still trying to parse together an overview of the underlying interactions to understand the ultimate physiological relevance. In this study, we unambiguously demonstrate a correlation between lowered growth temperature and concomitant relative increase in the amount of unsaturated hydrocarbons in *Synechocystis*. We provide experimental and literature-based evidence to support our hypothesis that hydrocarbons may play a role in adjusting the thylakoid membrane dynamics to compensate for decreased membrane fluidity under suboptimal temperatures. The proposed mechanism is analogous to the common fluidity effect induced by membrane lipid desaturation but confined to local parts of the thylakoid membrane to aid lateral diffusion and interactions of the integral photosynthetic complexes. Although many questions still need to be answered, the work provides a new pieces in the puzzle toward the elucidation of the ultimate function(s) of hydrocarbons in photoautotrophic cyanobacteria.

## Data Availability Statement

The original contributions presented in the study are included in the article/Supplementary Material, further inquiries can be directed to the corresponding authors.

## Author Contributions

Conceptual design and background work by EV, KT, and PK. Experimental work by EV and KT, except Δ*ado* strain preparation by JK, MIMS analysis by DF, chlorophyll fluorescent analysis by TH, and statistical evaluation of hydrocarbon production by HD. Result evaluation, data interpretation and drafting of the manuscript by all authors. Finalization of the manuscript and the printed figures by EV and PK. All authors contributed to the article and approved the submitted version.

## Funding

This work has been funded by Tekes LiF project (#40128/2014), the Academy of Finland CoE project “Molecular Biology of Primary Producers” (#307335), the Nordforsk Nordic Center of Excellence program “NordAqua” (#82845), and Jane and Aatos Erkko Foundation (#4605–26422).

## Conflict of Interest

The authors declare that the research was conducted in the absence of any commercial or financial relationships that could be construed as a potential conflict of interest.

## Publisher’s Note

All claims expressed in this article are solely those of the authors and do not necessarily represent those of their affiliated organizations, or those of the publisher, the editors and the reviewers. Any product that may be evaluated in this article, or claim that may be made by its manufacturer, is not guaranteed or endorsed by the publisher.
