# Supplementary material for: Hydrocarbon Desaturation in Cyanobacterial Thylakoid Membranes Is Linked With Acclimation to Suboptimal Growth Temperatures
Source: Front Microbiol. 2021 Nov 26;12:781864. doi: 10.3389/fmicb.2021.781864 (PMC8661006; doi:10.3389/fmicb.2021.781864)
Supplement: Supplementary file 1 [file Data_Sheet_1.PDF]

SUPPLEMENTARY MATERIAL for Vuorio et al 2021

[Hydrocarbon desaturation in cyanobacterial thylakoid membranes is linked with acclimation to suboptimal growth temperatures by Vuorio E., Thiel K., Fitzpatrick D., Huokko T., Kämäräinen J., Dandapani H., Aro E-M. and Kallio P.]

Fig. S1 GC-MS analysis of intracellular aliphatic hydrocarbons produced by *Synechocystis* p.2
Fig. S2 Localization of aliphatic hydrocarbons between insoluble and soluble cell fractions p.3
Fig. S3 Growth curves of WT *Synechocystis* at 15°C, 22°C, 30°C and 38°C p.4
Fig. S4 Absorbance spectra (400 nm – 750 nm) of *Synechocystis* on day 8 at different temperatures p.5
Fig. S5 Statistical evaluation of hydrocarbon saturation in respect to temperature p.6
Fig. S6 Verification of the ADO antibody using different *Synechocystis* fractions p.7
Fig. S7 Quantitative comparison between the total hydrocarbon content and ADO expression p.8
Fig. S8 PCR verification of the complete segregation of the *Synechocystis ado*::KmR (Δ*ado*) p.9
Fig. S9 Growth curves of *Synechocystis* WT and Δ*ado* grown at 22 °C and 30 °C in MC1000 p.10
Fig. S10 Resuspension of sedimented *Synechocystis* WT and Δ*ado* cells p.11
Fig. S11 Chlorohlyll fluorescence analysis (Dual-PAM-100) of *Synechocystis* WT and Δ*ado* p.12
Fig. S12 Fluorescent state transition analysis for WT and Δ*ado* strains grown at 22°C and 30°C p.13

Table S1 List of PCR primers used in this work p.14
Table S2 Correlation analysis between cultivation temperature and hydrocarbon saturation p.15

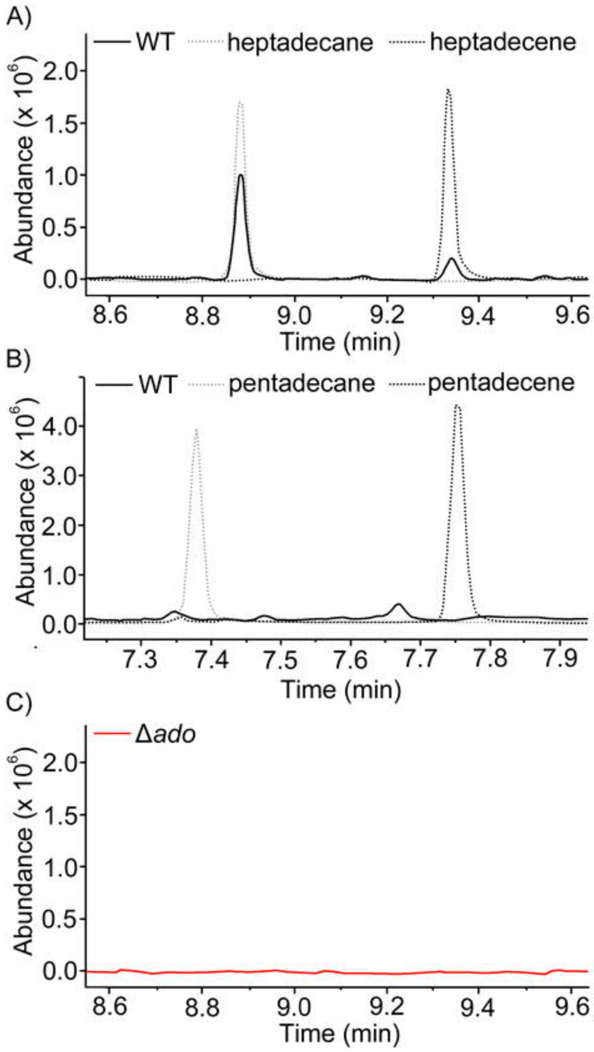

Figure S1: GC-MS analysis of intracellular aliphatic hydrocarbons produced by WT *Synechocystis* sp PCC 6803 (black line). The samples were analyzed for **A)** heptadecane and heptadecene and **B)** pentadecane and pentadecene based on corresponding C15 and C17 commercial standards (dotted lines).**C**) The target hydrocarbons were not detected in the Δ*ado* strain (red line).

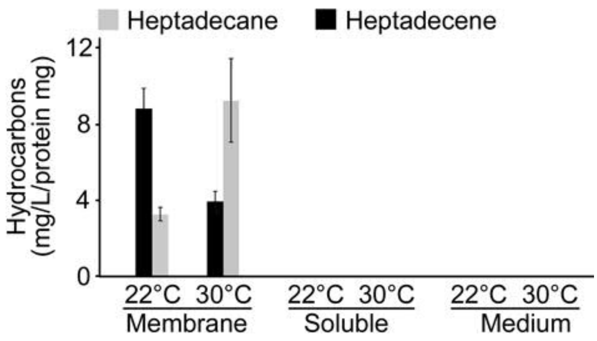

Figure S2: Localization of aliphatic hydrocarbons between insoluble and soluble cell fractions. The target hydrocarbons, heptadecene (black bars) and heptadecane (grey bars), analysed from the membrane fraction, soluble fraction, and the culture medium of WT *Synechocystis* sp. PCC 6803 cultivations incubated at 22 °C and 30 °C for four days. The averages and standard deviations were calculated based on three independent replicates.

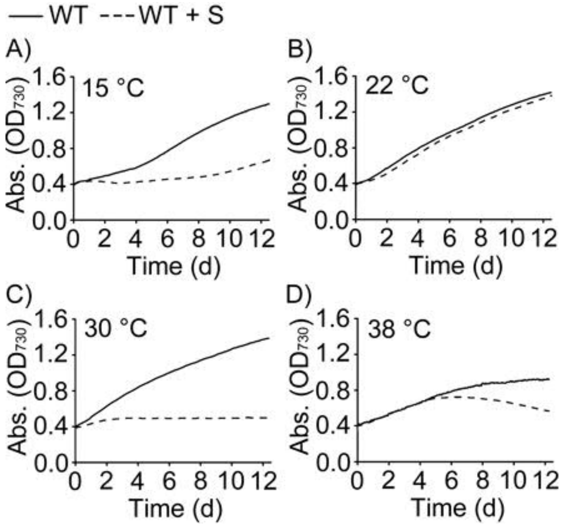

Figure S3: Representative growth curves (out of four parallel biological replicates) of WT *Synechocystis* sp. PCC 6803 cultivated at different temperatures under continuous light 50 µmol photons m^{-2} s^{-1}. The cells were cultured at **A)** 15 °C, **B)** 22 °C, **C)** 30 °C, **D)** 38 °C in standard BG11 (solid line) and with supplied 0.5 M sorbitol (dashed line), and monitored in photobioreactor MC1000 for 12 days.

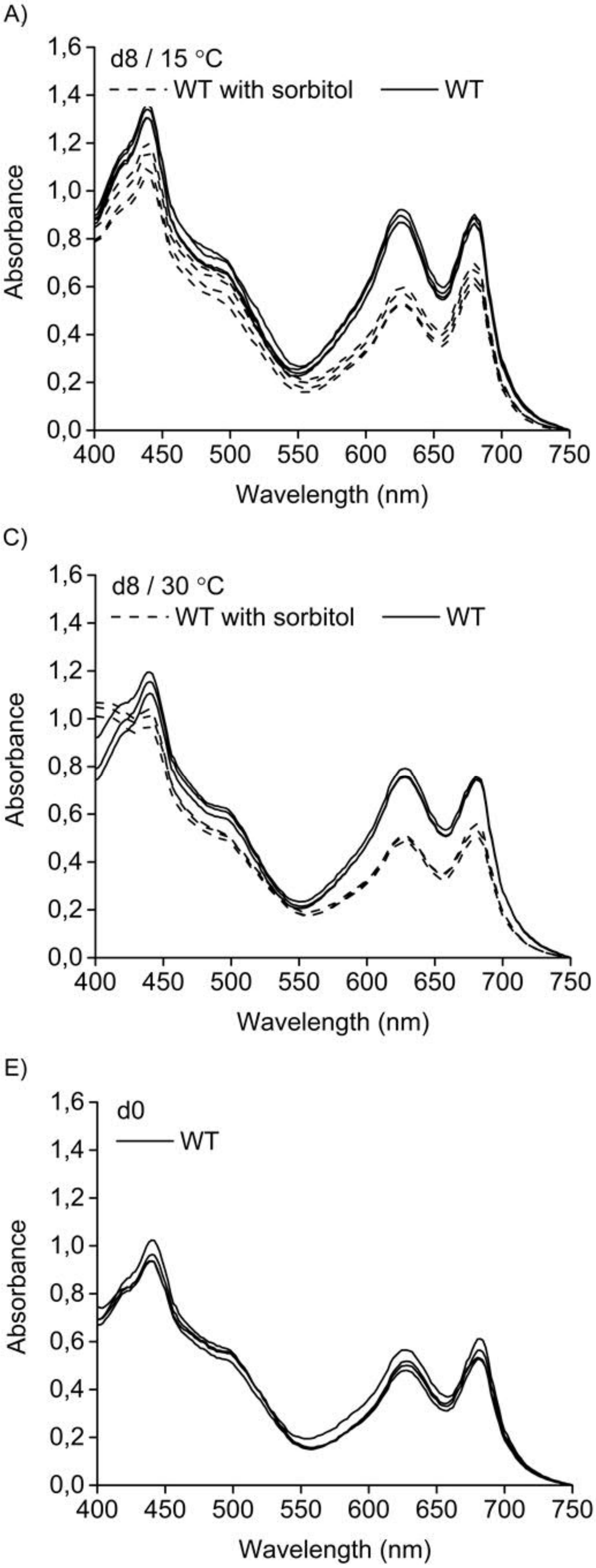

Figure S4: Absorbance spectra (400 nm – 750 nm) of WT *Synechocystis* sp. PCC 6803 cells cultured under osmotic stress induced with 0.5 M sorbitol (dashed lines) and without osmotic stress (solid lines) for eight days in the photobioreactor MC1000 at **A)** 15 °C, **B)** 22 °C, **C)** 30 °C and **D)** 38 °C and at **E)** the time of inoculation. Each graph is an overlay of three parallel independent replicates normalized to 750 nm

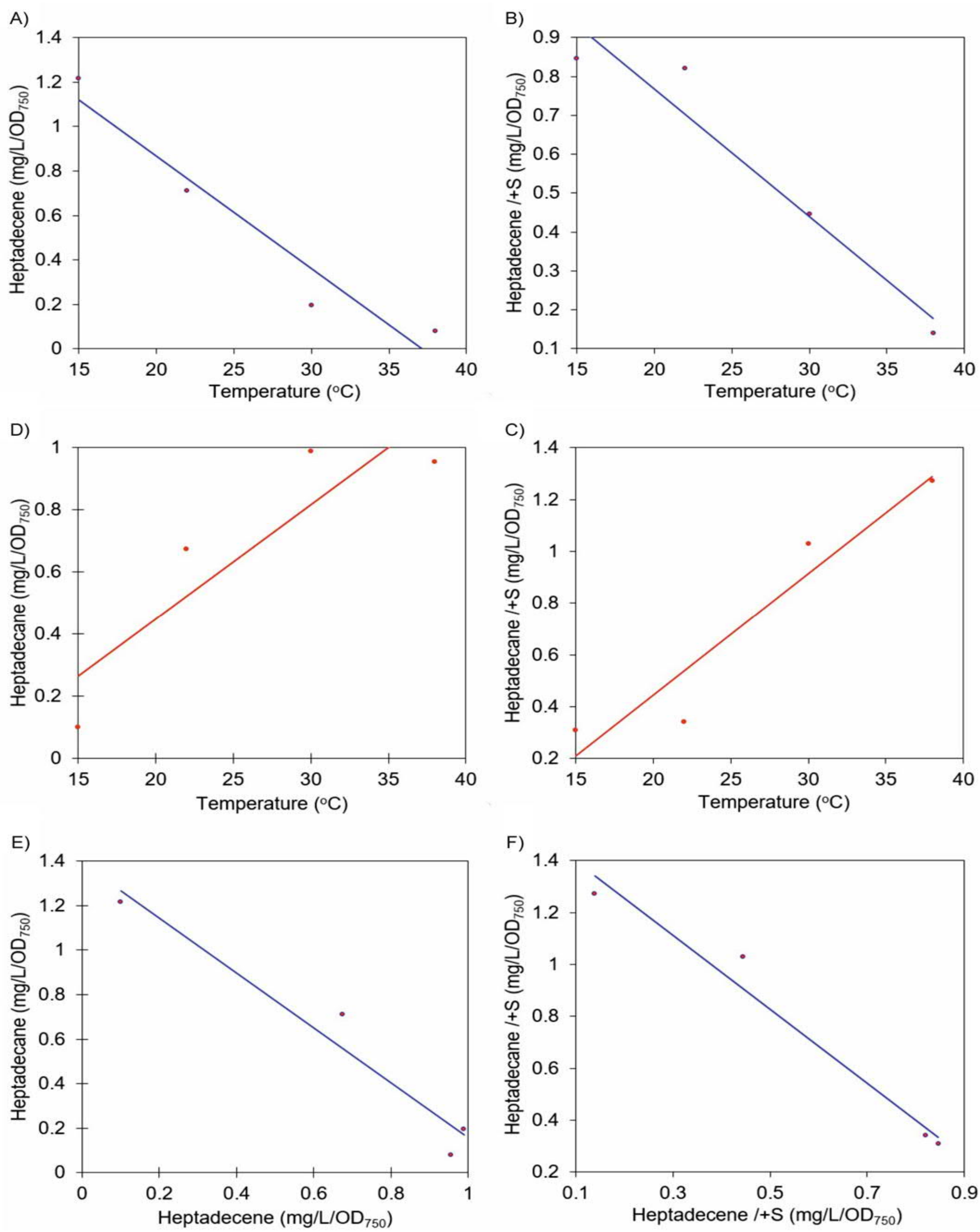

Figure S5: Statistical evaluation of hydrocarbon saturation in respect to temperature, based on the data collected on cultivation day 8 as presented in Figure 2. Regression analysis between the cultivation temperature and the amount of intracellular **(A)** heptadecene, **(B)** heptadecene (+S; cells grown in the presence of sorbitol), **(C)** heptadecane and **(D)** heptadecane (+S; cells grown in the presence of sorbitol) in WT *Synechocystis* sp. PCC 6803. Regression analysis between the amount of heptadecane and heptadecene measured in cells cultured **(E)** in the absence of sorbitol and **(F)** in the presence of sorbitol (+S).

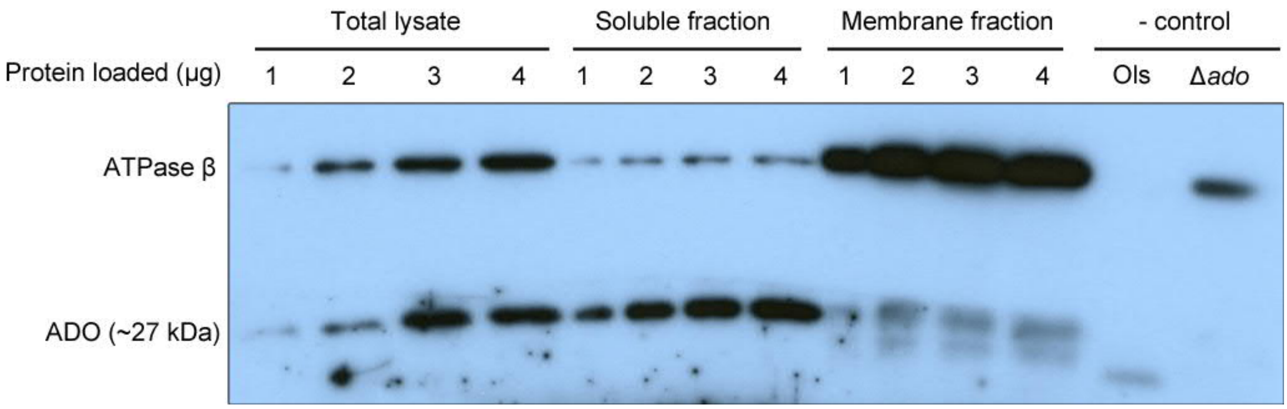

Figure S6: Verification of the antibody generated for *Synechocystis* sp. PCC 6803 ADO (calculated molecular weight ~ 27 kDa) by Western blot analysis. Different concentrations (1-4µg of loaded protein) of unfractionated total cell lysate, soluble supernatant fraction and membrane fraction were subjected to SDS-PAGE, and blotted with protein-specific antibodies against ADO and α-ATPase β. The negative controls (- control) were total protein extracts from *Synechococcus* sp. PCC 7002 harboring the Ols pathway, and the *Synechocystis* Δ*ado* strain.

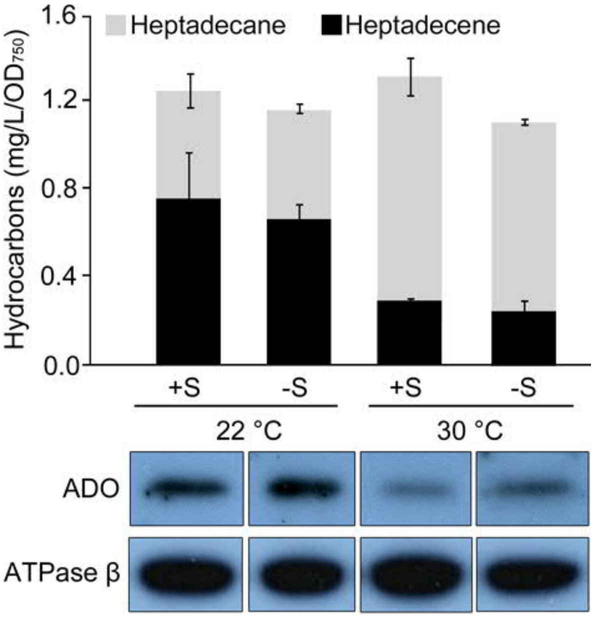

Figure S7: Quantitative comparison between the total hydrocarbon content (top) and ADO expression (bottom) in *Synechocystis* sp. PCC 6803 after two-day cultivation under 22 °C and 30 °C with 0.5 M sorbitol (+S) and without sorbitol (-S). The total hydrocarbon content is presented in the bar chart as the sum of heptadecene (black bar) and heptadecane (grey bar) quantitated by GC-MS. The averages and standard deviations were calculated based on three independent replicates. The relative amount of ADO present under each condition was analyzed by Western from unfractionated samples (3 µg of total protein loaded), using α-ATPase β as reference. The Western blot analysis was conducted in three replicates (representatives shown).

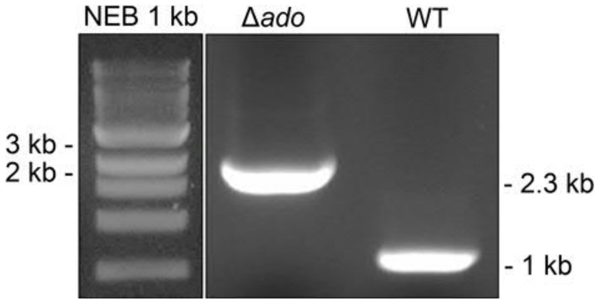

Figure S8: PCR verification of the complete segregation of the *Synechocystis ado*::KmR (Δ*ado*) as visualized by agarose gel electrophoresis. The expected sizes of the PCR fragments were ~2.3 kb for Δ*ado* and ~1 kb for the WT *Synechocystis* sp. PCC 6803.

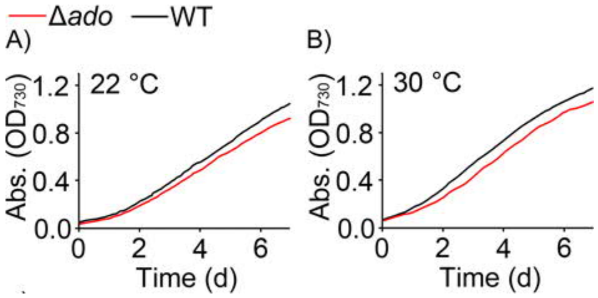

Figure S9: Growth curves of *Synechocystis* sp. PCC 6803 WT (solid black line) and Δ*ado* (solid red line) at **A)** 22 °C and **B)** 30 °C monitored in photobioreactor MC1000 for 7d in continuous light 50 µmol photons m^{-2} s^{-1}. The curves are representatives of three parallel biological replicates.

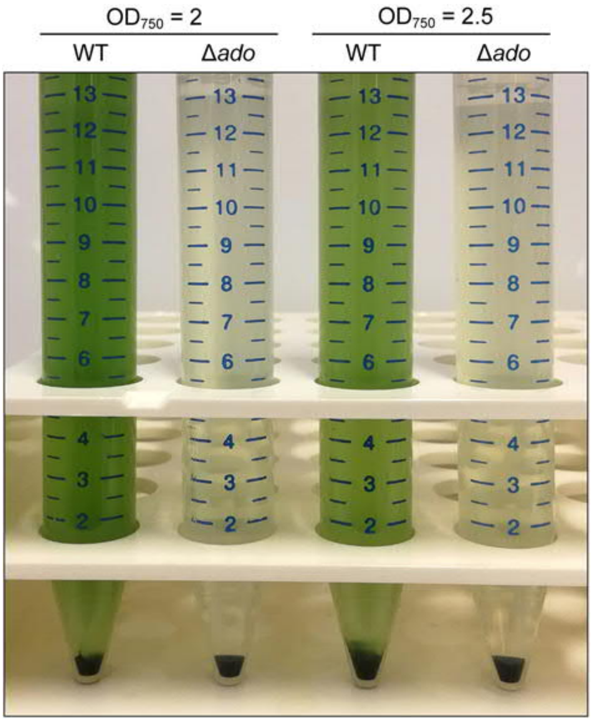

Figure S10: Comparison of the resuspension properties of sedimented *Synechocystis* WT and Δ*ado* cells. The samples were photographed after the cells had completely sedimented over an eight-day incubation at RT (c.f. Figure 4), followed by vigorous shaking and vortexing for about 30 seconds.

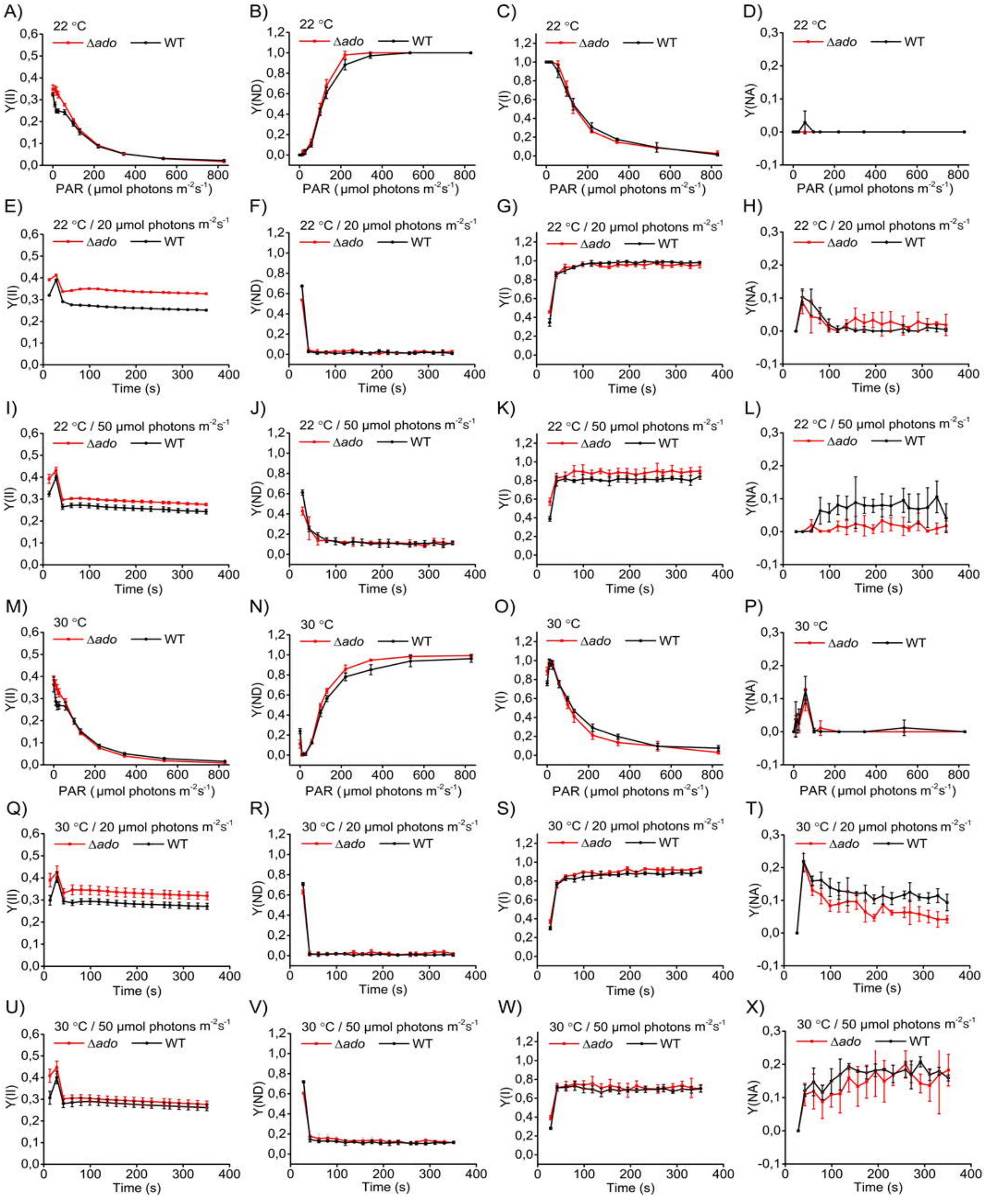

Figure S11. Photosynthetic activity of *Δado* and WT *Synechocystis* sp. PCC 6803 strains grown for four days at **A-L)** 22 °C and **m-y)** 30 °C was studied in detail by using light induction curve (**A-D and M-P**) in addition to two induction light, 20 µmol photons m^{-2} s^{-1} (**E-H and Q-T**) and 50 µmol photons m^{-2} s^{-1} **(I-L and U-X)**. The measured parameters included PS II activity (**A, E, I, M, Q, U**), donor side limitation **(B, F, J, N, R, V)**, PS I activity (**C, G, K, O, S, W**) and acceptor side limitation (**D, H, L, P, T, X**). Black lines represent WT *Synechocystis* and red *Δado*. The samples were normalized to Chl *a* = 15 µg ml^{-1} and three biological replicates were used to calculate the averages and standard deviations

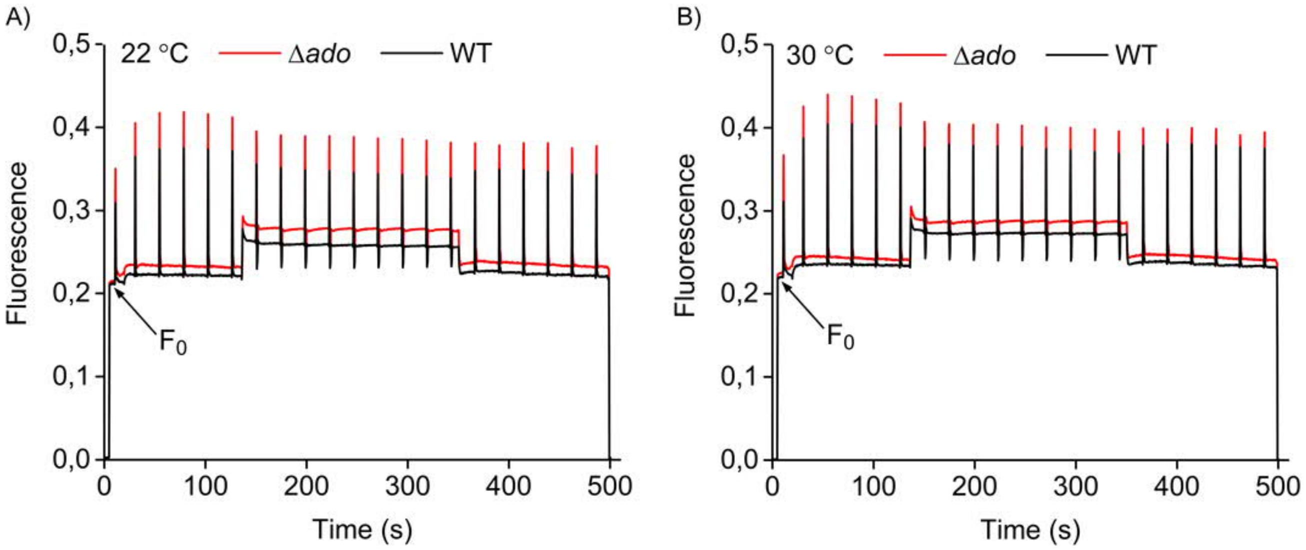

Figure S12. State transitions were induced by changing the light from blue to red and back to blue. The cultures were grown at **A)** 22 °C and **B)** 30 °C for four days and the black lines represent WT *Synechocystis* sp. PCC 6803 and red *Δado*. The samples were normalized to Chl *a* = 15 µg ml^{-1} and the averages and standard deviations were calculated based on three biological replicates.

SUPPLEMENTARY TABLES

Table S1: PCR primers used in the work

Primer name	Sequence 5’-3’
Fwd_*sll0208*-SacI	TCCCCCCCAGCAACTTAGACTAGTT
Rev_*sll0208* BamHI	GAAATAGTTGAAGATATTTCTAGCCGGGA
Km^R-NcoI-for	ATATATCCATGGATGCCTATTTGTTTATTTTTCTAAATACATTCAAATATGTAT
Km^R-NcoI-rev	ATATATCCATGGCTGAGCAATAACTAGCATAACCC
Fwd_*sll0209*-SacI	TATCGAGCTCCCACAAATCCCAAGCTTCTTAGG
Rev_*sll0209*-SphI	GTATGCATGCCTCTTTGAGCTAGCCCAGC
kan_AvrII-Fwd	ATATCCTAGGATGCCTATTTGTTTATTTTTCTAAATACATTCAAATAT
kan_AvrII-Rev	ATATCCTAGGCTGAGCAATAACTAGCATAACCCCTT

Table S2: Correlation analysis between cultivation temperature and saturation of native hydrocarbons heptadecane and heptadecene in *Synechocystis* sp.PCC 6803 cultivated **A)** in the absence of sorbitol and **B)** in the presence of sorbitol. The analysis is based on the data collected on cultivation day 8 presented in Figure 2.

A)

**Variables**	**Correlation**	**P-values**
Temperature vs Heptadecane	0.858	0.142
Temperature vs Heptadecene	-0.972	0.028
Heptadecane vs Heptadecene	-0.935	0.065

B)

**Variables**	**Correlation**	**P-values**
Temperature vs Heptadecane (cells supplemented with Sorbitol)	0.956	0.044
Temperature vs Heptadecene (cells supplemented with Sorbitol)	-0.966	0.034
Heptadecane vs Heptadecene (cells supplemented with Sorbitol)	-0.985	0.015
